# High glucose-upregulated PD-L1 expression through RAS signaling-driven downregulation of PTRH1 leads to suppression of T cell cytotoxic function in tumor environment

**DOI:** 10.1186/s12967-023-04302-4

**Published:** 2023-07-11

**Authors:** Chenggang Gao, Jiaoshun Chen, Jianwei Bai, Haoxiang Zhang, Yanyi Tao, Shihong Wu, Hehe Li, Heshui Wu, Qiang Shen, Tao Yin

**Affiliations:** 1 Present Address: Department of Pancreatic Surgery, Union Hospital, Tongji Medical College, Huazhong University of Science and Technology, Wuhan, 430022 China; 2 Sino-German Laboratory of Personalized Medicine for Pancreatic Cancer, Union Hospital, Tongji Medical College, Huazhong University of Science and Technology, Wuhan, 430022 China; 3 Department of Hematology, Union Hospital, Tongji Medical College, Huazhong University of Science and Technology, Wuhan, 430022 China; 4 Louisiana State University Health Sciences Center, New Orleans, LA 70112 USA

**Keywords:** PD-L1, Diabetes, Pancreatic ductal adenocarcinoma, Tumor microenvironment, RNA binding protein

## Abstract

**Background:**

Nearly 80% of patients with pancreatic cancer suffer from glucose intolerance or diabetes. Pancreatic cancer complicated by diabetes has a more immunosuppressive tumor microenvironment (TME) and is associated with a worse prognosis. The relationship between glucose metabolism and programmed cell death-Ligand 1 (PD-L1) is close and complex. It is important to explore the regulation of high glucose on PD-L1 expression in pancreatic cancer and its effect on infiltrating immune effectors in the tumor microenvironment.

**Methods:**

Diabetic murine models (C57BL/6) were used to reveal different immune landscape in euglycemic and hyperglycemic pancreatic tumor microenvironment. Bioinformatics, WB, iRIP [Improved RNA Binding Protein (RBP) Immunoprecipitation]-seq were used to confirm the potential regulating role of peptidyl-tRNA hydrolase 1 homolog (PTRH1) on the stability of the *PD-L1* mRNA. Postoperative specimens were used to identify the expression of PD-L1 and PTRH1 in pancreatic cancer. Co-culturing T cells with pancreatic cancer cells to explore the immunosuppressive effect of pancreatic tumor cells.

**Results:**

Our results revealed that a high dose of glucose enhanced the stability of the *PD-L1* mRNA in pancreatic tumor cells by downregulating PTRH1 through RAS signaling pathway activation following epidermal growth factor receptor (EGFR) stimulation. PTRH1 overexpression significantly suppressed PD-L1 expression in pancreatic cells and improved the proportion and cytotoxic function of CD8^+^ T cells in the pancreatic TME of diabetic mice.

**Conclusions:**

PTRH1, an RBP, plays a key role in the regulation of PD-L1 by high glucose and is closely related to anti-tumor immunity in the pancreatic TME.

**Supplementary Information:**

The online version contains supplementary material available at 10.1186/s12967-023-04302-4.

## Background

Pancreatic cancer, one of the most refractory malignancies, has very few surgical opportunities and a high recurrence rate after resection [1]. Nearly 80% of patients with pancreatic cancer experience glucose intolerance or diabetes [2], further complicating the treatment. Long-standing diabetes (onset > 36 months before the neoplastic diagnosis) is considered as a risk factor for pancreatic cancer [3], and patients with pancreatic cancer complicated by diabetes have a worse prognosis [4]. Pancreatic cancer incidence increases linearly correlated with increasing fasting glucose levels, even in populations with normal glucose range [5]. A high dose of glucose induces epidermal growth factor (EGF) expression and transactivates EGF receptor (EGFR) [6], which can drive programmed death 1 ligand 1 (PD-L1) expression via downstream effector pathways [7]. In one retrospective analysis [8], 19% of pancreatic cancer samples had upregulated PD-L1, indicating lymphocyte exhaustion and showing an association with poorer disease-free and overall survival.

Non-coding regions [5′- and 3′-untranslated regions (UTRs)], which contain *cis*-regulatory elements, play an important role in regulating its transcription. Kogure et al. reported removal of the *PD-L1* 3′-UTR significantly enhances its expression in different human cancers [9]. RNA binding proteins (RBPs) are a class of proteins involved in splicing, modifications, transport, localization, stability, degradation, and translation of RNAs [10]. Diverse RBPs have been identified as potential regulatory factors affecting *PD-L1* mRNA stability using the flag-peptide-tagged RNA pull-down method [9]. As an essential post-transcriptional regulation of PD-L1 expression, the potential immunoregulatory effects of multiple RBPs in several cancers, such as breast cancer [11, 12] and non-small cell lung cancer [13, 14], have been revealed. However, the role of RBPs in regulating PD-L1 in pancreatic cancer remains unknown, especially in the context of high glucose. In this report, we investigated the mechanisms underlying to the immunosuppressive effect of high glucose levels in pancreatic cancer. Our study provides a basis for understanding the dynamic mechanism driving immune landscape pattern changes in the pancreatic tumor microenvironment (TME) and a potential intervention strategy to improve anti-tumor immunity.

## Methods

### Cell lines

Human PC cell lines PANC-1, AsPC-1, MIA PaCa-1, BxPC-3 and SW1990 were obtained from the American Type Culture Collection (Manassas, VA, USA). The Human PC cell lines CFPAC-1 and murine ductal pancreas adenocarcinoma cell line Panc-02 were obtained from Procell (Procell Bio-Tech, Shanghai, China). The absence of *Mycoplasma* contamination for all cell lines was confirmed by MycoBlue Mycoplasma Detector (Vazyme, Nanjing, China). Cells were cultured in Dulbecco’s Modified Eagle Medium (DMEM) of various glucose concentration complemented with 10% fetal bovine serum (Gibco Invitrogen, Grand Island, NY, USA) and 100 U/ml penicillin/streptomycin (Beyotime Biotechnology, Shanghai, China).

### RNA immunoprecipitation assay

Endogenous PTRH1 protein and negative control IgG were pulled down to obtain the RNA library from SW1990 cell line. RNA-binding protein immunoprecipitation (RIP) assay was performed using EZ-Magna RIP^™^ RNA-Binding Protein Immunoprecipitation Kit (Millipore #17-701) according to the manufacturer’s protocol. The expression level of *PD-L1* mRNA was detected by quantitative real-time PCR. Three independent experiment were performed.

### Improved RNA binding protein immunoprecipitation

Improved RNA binding protein immunoprecipitation was performed using the SW1990 cell line. Specifically, cells were cross-linked on ice with UV irradiation type C (254 nm) at 400 mJ/cm^2^ in the presence of cold PBS (4 ml per 15-cm dish). Cell lysis and immunoprecipitation experimental protocol were introduced on Ablife’s official website (https://www.ablife.cc). The cDNA libraries used the Illumina ScriptSeq^™^ v2 RNA-Seq Library Preparation Kit (Epicentre). The cDNAs were purified and amplified and PCR products corresponding to 200–500 bps were purified, quantified and stored at – 80 ℃ until used for sequencing. For high-throughput sequencing, the libraries were prepared following the manufacturer's instructions and applied to Illumina HiSeq X Ten system for 150 nt paired-end sequencing by ABlife. Inc (Wuhan, China). iRIP-seq data workflow is shown in Additional file 1: Fig. S1. Three independent strategies for binding peak analysis were performed following the methods mentioned in previous studies: Ablife [15], Piranha [16], and Cims [17].

### Co-culture T cells and pancreatic cancer cells

Isolation of mononuclear cells from fresh human peripheral venous blood was performed using Lymphoprep^™^ product according to the manufacturer’s protocol (Stemcell Technologies). Isolate untouched and highly purified naive pan T cells from fresh human peripheral blood mononuclear cells (PBMCs) was performed using EasySep^™^ referring to directions from Stemcell Technologies. T cells were activated by incubation with ImmunoCult^™^ Human CD3/CD28 T Cell Activator (Stemcell Technologies) and expanded over 14 days adding IL-2. Activated T cells were adding to the well after pancreatic cell adhering to the wall (E:T = 1:1). The production of IFN-γ in CD8^+^ T cells was detected by flow cytometry after 48 h co-culture with differently treated pancreatic cells. Three independent samples were detected in each group.

### Cell transfection and lentivirus infection

The PTRH1 siRNA, STAT3 siRNA, and NC siRNA were designed and synthesized by Ribobio (Guangzhou, China). The PTRH1-shRNA and PTRH1-overexpression plasmid were designed and synthesized by ABlife. The siRNA and shRNA sequences are shown in Additional file 14: Table S1. Lipofectamine^™^2000 (Invitrogen, CA, USA) was used for cell transfection followed the manufacturer’s protocol. Nonsilencing (NC)‐shRNA lentivirus (puromycin resistance) and PTRH1‐overexpression lentivirus (puromycin resistance) were purchased from GeneChem (Shanghai, China). Stable cell lines were selected with 0.5 μg/ml puromycin (PTRH1 oe cells) for 2 days.

### Lactate concentration and glucose concentration measurements

Lactate measurements of tumors in euglycemic mice (n = 8) and hyperglycemic mice (n = 8) were performed using the lactic acid assay kit (Nanjing Jiancheng Bio, Nanjing, China, #A019-2-1) following manufacturer’s protocol and standardized by the total protein content in each sample. Glucose concentration in tumor tissues was determined using the Glucose Content Assay Kit (Solarbio, Beijing, China, #BC2505), following manufacturer’s protocol and standardized by the total protein content in each sample.

### Diabetic murine models

All experiments involving animals in this research followed the ethical standards set by the Institutional Animal Care and Use Committee of Tongji Medical College, Huazhong University of Science and Technology. Five-week-old male C57BL/6 mice were purchased from China Three Gorges University. For the diabetes group, mice were injected for 5 consecutive days with 50 mg/kg streptozocin (STZ) (Sigma, St. Louis, MO, USA, #S0130) dissolved in cold fresh sodium citrate buffer (pH = 4.5). Sannuo glucometer (Sannuo, Changsha, China) was used to measure the glucose levels in blood samples taken from the tail vein. The diabetic murine models were considered successful when their blood glucose continued ≥ 11 mM.

### Orthotopic mouse pancreatic cancer models

Panc-02 cells transfected with NC, OE-PTRH1 lentivirus that were injected into the pancreas tail of C57BL/6 mice by laparotomy. Mice were anesthetized using 1.25% tribromoethanol intraperitoneal injection (20 μl/g mice). The tumors were created by injecting 1 × 10^6^ cells in 10 μl PBS^+^ 10 μl Matrigel in the tail of the pancreas through the left-flank incision when the mice blood glucose reached the standard of diabetes (> 11.1 mmol/l). Half of each group were given Anti-PD-L1 treatment 10 mg/kg once every 2 days for total 4 intraperitoneal injections during 4th week. The implanted orthotopic pancreatic tumor were harvested at 28th day (Additional file 2: Fig. S2).

### mRNA half-life assay

Cells transfected with PTRH1 siRNA or ncRNA were treated with actinomycin D (Sellect, USA, #S8964) 5 μg/ml. Actinomycin D can be absorbed by cells within minutes and preferentially embedded into GC-rich DNA sequences to form stable complexes that inhibit the transcription process of all eukaryotic RNA polymerases. After treatment with actinomycin D for 0, 1, 2, 4, 6, and 8 h, the level of *PD-L1* mRNA was detected by RT-PCR. 18S RNA was detected as control and the sequence of primers used is shown in Additional file 14: Table S1. Three independent experiment were performed.

### Human pancreatic cancer and paracancer specimens

Human pancreatic cancer (n = 16) and paracancer specimens (n = 16) were collected after surgery from pancreatic cancer patients admitted to Wuhan Union Hospital from March to September 2020. Informed consent was obtained from patients for all samples. Immediately after the sample is cut, it is stored in liquid nitrogen. The patient was diagnosed with diabetes after two fasting blood glucose indices greater than 7 mmol/l. Sixteen cases of paired pancreatic ductal adenocarcinoma (PDAC) and adjacent tissue samples were collected. Seven of the patients had a context in diabetes and the others had no history of diabetes. There is no statistically significant difference in age or gender between patients with and without diabetes.

### Flow cytometry analysis

Single-cell suspensions of tumor-infiltrated immune cells were prepared. Cells were incubated with anti-CD16/CD32 antibody (BD Pharmingen, #553141) to minimize non-specific binding. All cell surface reactions (CD45, CD3, CD4, CD8, CD25, CD206, I-A/I-E, CD11c, CD11b, F4/80, PD-1, CD86, Ly-6G, and Ly-6C) were performed at 4 ℃ for 30 min. Activated human primary T cells (Stemcell Technologies) were treated for 24 h and 4 h, respectively, with a PMA/ionomycin mixture (MultiSciences, Hangzhou, China, #70-CS1001) and brefeldin A (BFA) (Sigma-Aldrich, MO, USA, #B5936) as pretreatment. The T cells were fixed and permeabilized using the Fixation/Permeabilization Set protocol (eBioscience, CA, USA, #00-5123-43, #00-5223-56, #00-8333-56) and incubated with IFN-γ antibody (BD Pharmingen, #554700) in the dark for 30 min. The permeabilization step allowing intracellular staining (KI-67, IFN-γ, GZMB, and Foxp3) was performed referring to the manufacturer’s protocol (BD Pharmingen, #562574, #550583). The gating strategy is shown in Additional file 3: Fig. S3. All the tumors in each group were detected. In addition, when using flow cytometry to measure the abundance of PD-L1 expression on the surface of pancreatic cancer cells, each group examined three independent samples.

### Quantitative real-time PCR assay

RNA was extracted from pancreatic cells treated with different concentration of glucose using TRIzol reagent (Invitrogen, USA), and cDNA was synthesized using a PrimeScript RT kit (Takara, Japan). cDNA was added to TB Green^™^ Fast qPCR Mix (Takara, Japan) for quantitative real-time PCR (qRT-PCR; Bio-Rad, USA). Quantitative real-time PCR (qRT-PCR) was performed as previously described [18]. The data was interpreted using the 2^−ΔΔCT^ method. The primer sequences are provided in the Additional file. Three independent experiment were performed.

### Western blot analysis

Pancreatic cancer cells and tumor tissues were lysed with RIPA lysis buffer (25 mM Tris–HCl pH 7.6, 150 mM NaCl, 1% NP-40, 1% sodium deoxycholate, 0.1% SDS) (Beyotime, China) supplemented with 1% protease inhibitors. Protein concentrations were determined using BCA Protein Assay Kit (Solarbio, China). Western blot was performed as previously described [19]. Details of the primary antibodies used are provided in the supplementary materials. Three independent experiment were performed.

### Immunohistochemistry (IHC) analysis

Hematoxylin staining and evaluation of the tissue architecture was used to define the stromal type. The tumors were stained with the following antibodies: PD-L1 (Cell Signaling, dilution 1:500), PTRH1 (BIOSS, dilution 1:200). Expression of PD-L1 was evaluated by counting specific cytoplasmic staining cells in 5 randomly selected areas at 40 × light for each group. The average immunity group image gradation analysis integral light density of PTRH1 in 5 randomly selected areas for each group were calculated by using Imagepro Plus 6.0 (Media Cybernetics, Inc).

### Bioinformatics

#### Single cell RNA-seq data processing

Pancreatic cancer single cell transcriptome sequencing data [PDAC samples complicated with diabetes (n = 10) and those without diabetes (n = 14)] were downloaded from Genome Sequence Archive (GSA) under project PRJCA001063, the accession number for the sequencing data is GSA: CRA001160. Preliminary identification of cell type was performed in R (v3.6.3) using SingleR (v1.8.1) toolkit with Human Primary Cell Atlas as reference data set. Cells attached different labels were then separately imported into the Seurat (v3.2.2) R toolkit for quality control. Analysis process were run with default parameters, unless specified otherwise. We characterized cell types of these clusters based on their highly expressed genes and their expression level of known markers (Additional file 4: Fig. S4, Additional file 5: Fig S5). Clusters with deficiency of characteristic markers for all cell types or presentence of characteristic markers for more than one cell type were excluded from further analysis. The differential expressed genes (DEG) were identified by org.Hs.eg.db package (v3.6.0) (Additional file 15: Table S2).

### The cancer genome atlas (TCGA) data analysis

Transcriptome sequencing data of pancreatic cancer were downloaded through the download tool GDC provided by the official TCGA. Differentially expressed genes were calculated by DESeq2 Significant differential genes were determined by the threshold of |log2FoldChange|≥ 0.5 and adjust *P value* ≤ 0.01. GO/KEGG enrichment was performed using the limma package (v3.36.5), clusterProfiler package (v3.8.1), and the org.Hs.eg.db package (v3.6.0) of R. Correlations between mRNA expression of RBPs and PD-L1 were tested using the Hmisc package (v4.2.0) of R, based on Spearman’s correlation coefficient.

### Statistical analysis

Statistical calculations were performed with R (v3.6.3). The results are shown as the mean ± SD. Comparisons between two groups or multiple groups were analyzed using Student’s t-test or one-way ANOVA. All tests were two-sided, a *p value* < 0.05 was considered statistically significant.

## Results

### High glucose inhibits T cell function in the TME by enhancing PD-L1 expression in pancreatic cancer cells

**Fig. 1:**
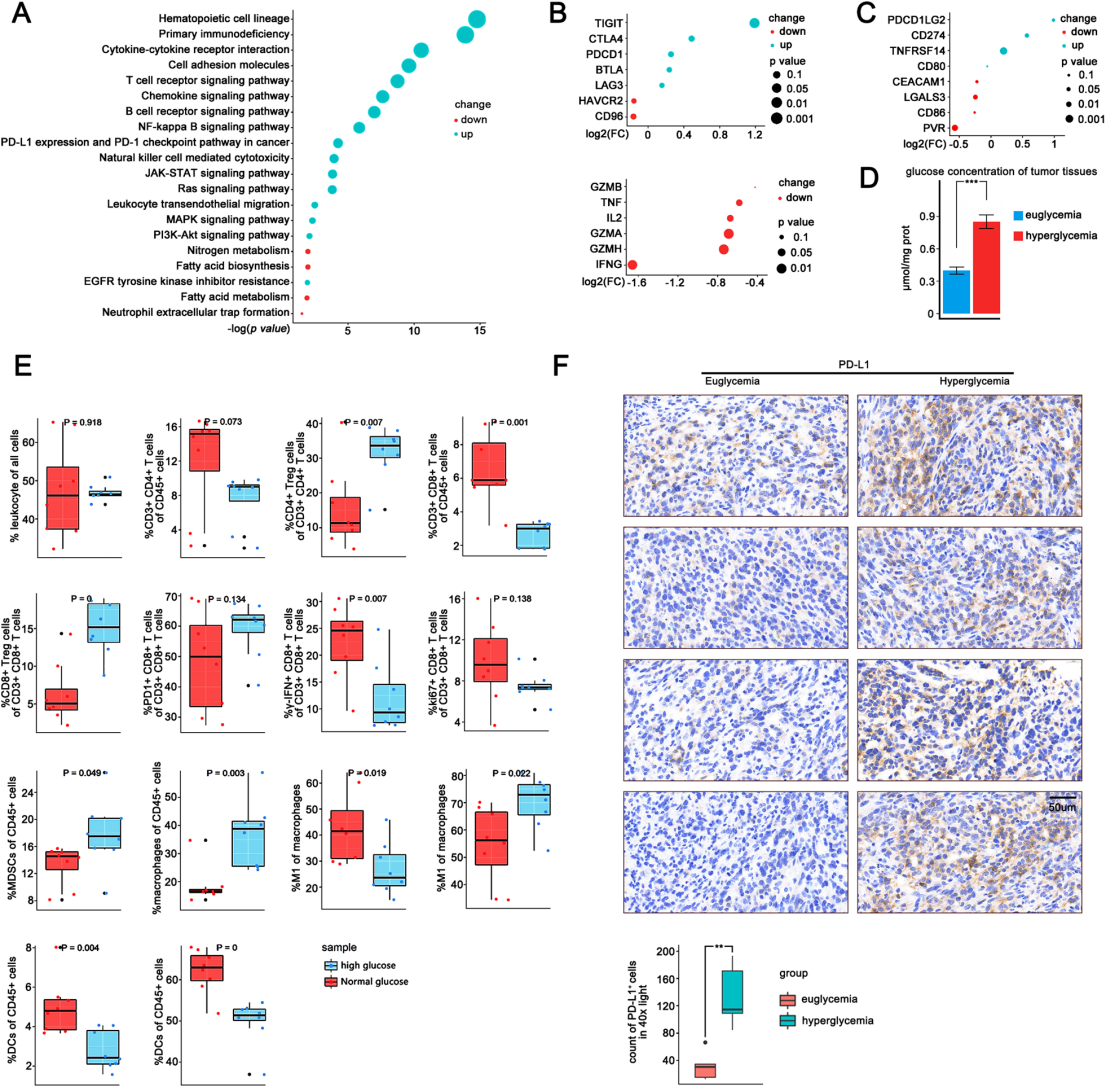
High glucose may inhibit T cell function in the tumor microenvironment by enhancing PD-L1 expression in pancreatic cancer cells. **A** KEGG pathway enrichment analysis of 146 PDAC samples from TCGA. Bubble charts depicting pathway changes in PDAC samples complicated with diabetes (n = 38) compared to samples without diabetes (n = 108). **B** Single cell transcriptome data of 24 pancreatic cancer tumors from a public cohort were analyzed (CRA001160). Bubble charts depicting differential genes related to immune checkpoints and killing function between CD8^+^ T cells in PDAC samples complicated with diabetes (n = 10) and samples without diabetes (n = 14). **C** Single cell transcriptome data of 24 pancreatic cancer tumors from a public cohort were analyzed (CRA001160). Bubble charts depicting differential genes related to immune checkpoints ligands between tumor epithelial cells in PDAC samples complicated with diabetes (n = 10) and samples without diabetes (n = 14). **D** Barplot depicting glucose content of tumor tissue in euglycemic (n = 8) and hyperglycemic mice (n = 8). **E** Flow cytometry analysis of the infiltration of immune effectors (CD45^+^ cells, CD3^+^ T cells, CD3^+^ CD4^+^ T cells, Tregs, CD3^+^ CD8^+^ T cells, IFN-γ^+^ CD8^+^ T cells, PD-1^+^ CD8^+^ T cells, KI67^+^ CD8^+^ T cells, DC, MDSC, Macrophages, M1-type macrophages, M2-type macrophages) in the orthotopic tumors of hyperglycemia (n = 8) and euglycemia mice (n = 8). **F** Immunohistochemical staining to evaluate the distribution and level of PD-L1 expression in the orthotopic tumors of hyperglycemia and euglycemia mice

Through KEGG pathway enrichment analysis of transcriptome sequencing data of pancreatic cancer from TCGA database (n = 146), we found specific oncogenic signaling pathways significantly enriched as well as PD-L1 expression and PD-1 checkpoint pathway in samples of PDAC complicated with diabetes (n = 38) (Fig. 1A). We analyzed a publicly available single cell transcriptome sequencing data from Genome Sequence Archive containing 24 pancreatic cancers and found that the intratumoral CD8^+^ T cells of diabetic patients (n = 14) had higher expression of immune checkpoints such as *TIGIT*, *CTLA4*, and *PDCD1*, but lower levels of *IFNG* and *GZMA*, than those of non-diabetic patients (n = 10) (Fig. 1B), suggesting that CD8^+^ T cells tended to exhibit an exhausted condition with impaired tumoricidal functions in the presence of high glucose. In addition, we found higher *PD-L1* expression in pancreatic tumor cells in PDAC samples with diabetes than in those without (Fig. 1C). However, CD8^+^ T cell infiltration did not differ between samples with and without diabetes (Additional file 6: Fig. S6A, B).

We then established orthotopic pancreatic tumor models in both healthy C57BL/6 mice (n = 8) and STZ-induced hyperglycemic mice (n = 8), and investigated their differences in the abundancy and function states of several subsets of immune cells, including CD4^+^ T cells, CD8^+^ T cells, macrophages, dendritic cells (DCs), and myeloid-derived suppressive cells (MDSCs). Compared to those in euglycemic mice, tumors in hyperglycemic mice contained higher concentrations of glucose (Fig. 1D). We found substantially higher percentages of macrophages, MDSCs, and regulatory t cells (Tregs), but lower proportions of CD4^+^ and CD8^+^ T cells among CD45^+^ leukocytes in tumors of hyperglycemic mice (Fig. 1E). The proportion of IFN-γ positive cells was significantly lower for infiltrating CD8^+^ T cells in hyperglycemic mice than in euglycemic mice (*p value* = 0.007). In addition, a larger M2 type-to-M1 type macrophage ratio was observed in tumors with hyperglycemia. The proportions of total DCs and activated DCs were lower in tumors of hyperglycemic mice than in euglycemic mice. Immunohistochemical staining revealed that pancreatic tumor cells under hyperglycemia express higher levels of PD-L1 than those under euglycemic conditions, especially at the edge of the tumor and the area around the vessels (Fig. 1F).

### Pancreatic cancer cells cultured with high glucose inhibit T cell killing in vitro

**Fig. 2:**
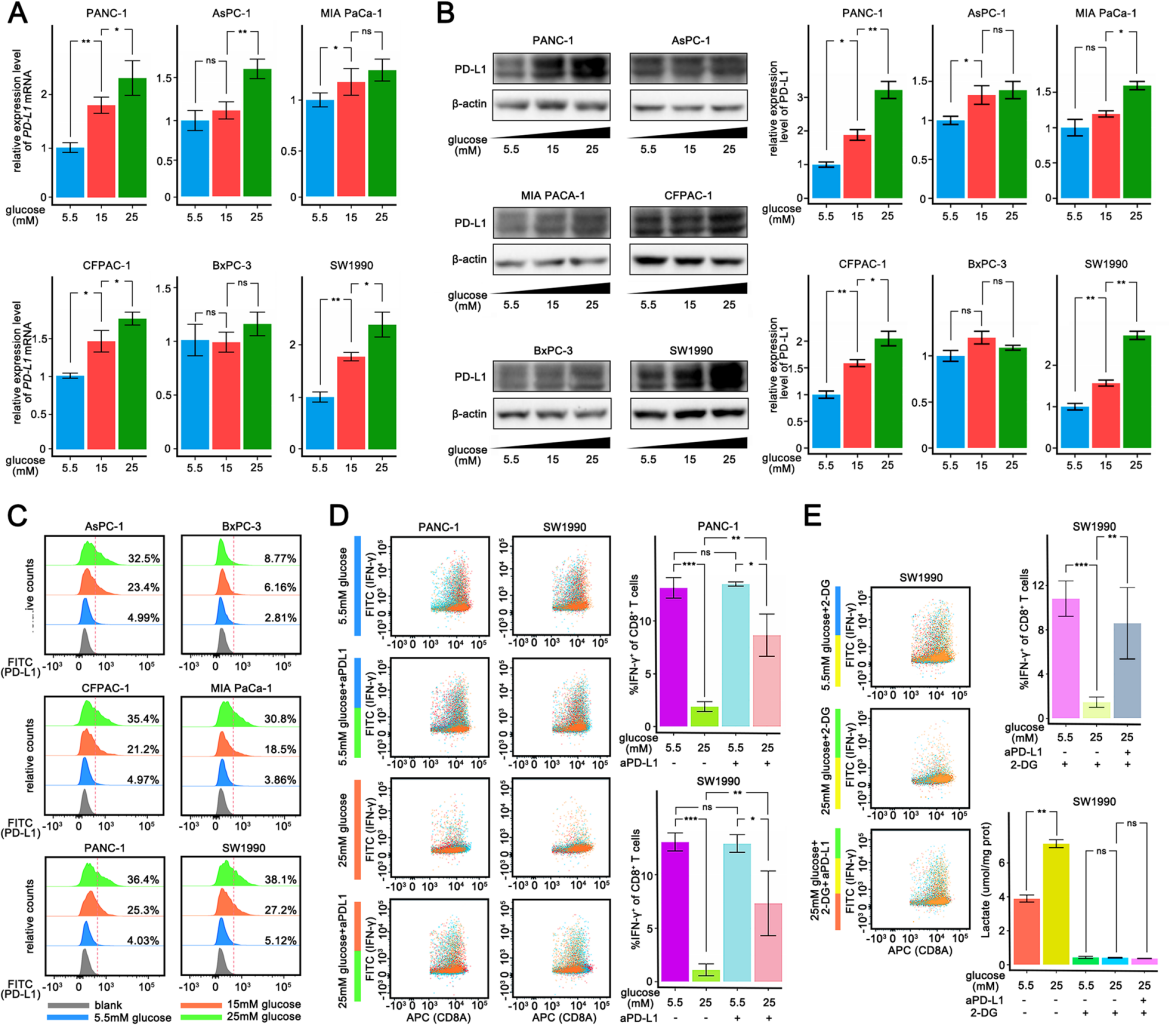
Pancreatic cancer cells cultured with high glucose inhibit T cell killing in vitro. **A**–**C** qPCR analysis** A** and Western blotting analysis **B** and Flow cytometry analysis** C** of *PD-L1* mRNA expression in PANC-1, AsPC-1, MIA PaCa-1, CFPAC-1, BxPC-3 and SW1990 cells 48 h after different sugar concentration (5.5 mM, 15 mM, 25 mM) medium culturing. **D** Flow cytometry analysis of IFN-γ production of cocultured CD8^+^ T cells with PANC-1 cells or SW1990 cells (E:T = 1:1) in different treatment. **E** Flow cytometry analysis of IFN-γ production of cocultured CD8^+^ T cells with PANC-1 cells or SW1990 cells (E:T = 1:1) cultured in different sugar concentration medium supplemented with 2-DG. *PDAC* pancreatic ductal adenocarcinoma, *TCGA* The Cancer Genome Atlas, *DC* dendric cell, *MDSC* myeloid-derived suppressor cell, *ns* non-significant. The graphs show representative results from three independently repeated experiments. *p  < 0.05, **p  < 0.01, ***p < 0.001

Then we cultured several pancreatic cell lines including PANC-1, AsPC-1, MIAPaCa-2, CFPAC-1, BxPC-3, and SW1990 at 5.5 mM, 15 mM, and 25 mM glucose concentrations for 48 h to explore the regulation of PD-L1 expression by glucose. The mRNA and protein levels of PD-L1 were up-regulated by glucose in a dose-dependent manner, except in BxPC3 cells (Fig. 2A, B and C). PANC-1 and SW1990 cells were then treated with different concentration of glucose (5.5 or 25 mM), and co-cultured with naïve T cells derived cytotoxic T cells (E:T = 1:1). Pancreatic cancer cells cultured in 25 mM glucose were more immunosuppressive against T cells (Additional file 7: Fig. S7), while the suppression was substantially diminished following anti-PD-L1 treatment (Fig. 2D). There was no effect on the secretion of IFN-γ by CD8^+^ T cells under high concentrations of glucose or PD-L1 antibodies to treat CD8^+^ T cells individually (Additional file 8: Fig. S8). It has been reported that high glucose levels may be involved in the functional inhibition of T cells owing to the enhancement of glycolysis in pancreatic cancer cells and the induced release of lactic acid [20]. To examine whether lactic acid was involved in T cell suppression, 2-DG was added to inhibit the glycolysis in pancreatic cancer cells, and subsequently erased the difference in lactate concentration between the two tumor groups of different glucose concentrations. However, the abundance of IFN-γ^+^CD8^+^ T cells were still lower in tumors treated with higher concentration of glucose (Fig. 2E). Notably, anti-PD-L1 treatment remained effective, increasing IFN-γ production in CD8^+^ T cells cocultured with PANC-1 cells. Collectively, we have demonstrated that high concentration of glucose upregulated PD-L1 expression in pancreatic cancer cells, and inhibited the tumoricidal function of T cells.

### High-concentration glucose upregulates PD-L1 in pancreatic tumor cells by activating EGFR downstream pathways

**Fig. 3:**
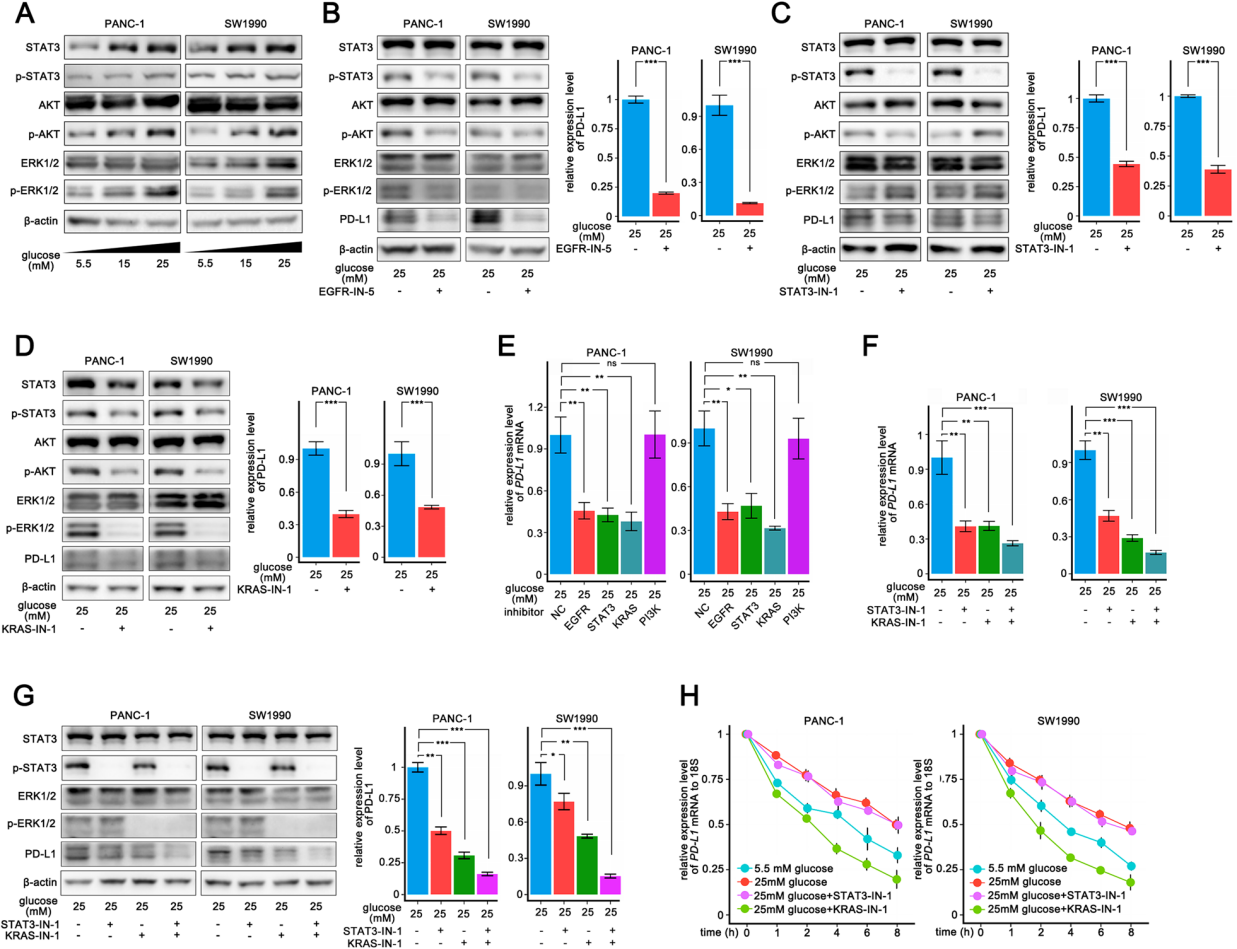
High concentration glucose upregulates PD-L1 expression of pancreatic tumor cells by activating EGFR downstream pathways**. A** Western blotting analysis of EGFR downstream pathways including RAS-ERK, STAT3, PI3K-Akt signaling in PANC-1 and SW1990 cells 48 h after different sugar concentration (5.5 mM, 15 mM, 25 mM) medium culturing. **B**–**D** Western blotting analysis of EGFR downstream signaling and PD-L1 expression in PANC-1 and SW1990 cells following 24 h treatment with EGFR-IN-5 **B** or STAT3-IN-1 **C** or KRAS-IN-1 **D** in 25 mM sugar medium. **E** qPCR analysis of *PD-L1* mRNA expression in PANC-1 and SW1990 cells following 24 h treatment with different inhibitors (EGFR-IN-5, STAT3-IN-1, KRAS-IN-1, PI3K-IN-1) in 25 mM sugar medium. **F** Western blotting analysis of PD-L1 expression in PANC-1 and SW1990 cells following 24 h treatment with STAT3-IN-1 or KRAS-IN-3 individually or combination of the two inhibitors in 25 mM sugar medium. **G** qPCR analysis of *PD-L1* expression in PANC-1 and SW1990 cells following 24 h treatment with STAT3-IN-1 or KRAS-IN-3 individually or combination of the two inhibitors in 25 mM sugar medium. **H** qPCR analysis of *PD-L1* mRNA stability in PANC-1 and SW1990 cells after the concomitant addition of actinomycin D (10 μg/ml) and STAT3-IN-1 or KRAS-IN-3 added at time = 0 h in 25 mM sugar medium. *ns* non-significant. The graphs show representative results from three independently repeated experiments. *p  < 0.05, **p  < 0.01, ***p  < 0.001

To investigate how high glucose concentration regulates PD-L1 expression, we first evaluated whether glucose regulates PD-L1 via the AMPK signaling pathway, which is considered to have low activity under high glucose concentration conditions and involved in immune escape [21]. We used a direct activator of AMPK signaling, 5-Aminoimidazole-4-carboxamide1-β-d-ribofuranoside (AICAR), in PANC-1 and SW1990 cell lines and did not found change in PD-L1 expression (Additional file 9: Fig. S9A). KEGG enrichment analysis of pancreatic adenocarcinoma (PAAD) samples from TCGA (Fig. 1A) showed that in pancreatic tumors with diabetes, the terms of EGFR downstream pathways, including JAK-STAT, RAS, and PI3K-Akt, were enriched. Number of studies examined the correlation between the EGFR or its downstream pathways and PD-L1 [22–24]. High concentration of glucose is considered to be effective activators of EGFR [6]. As expected, high concentrations of glucose led to a stronger EGFR signal; consequently, p-STAT3, p-ERK, and p-Akt were upregulated (Fig. 3A). EGFR inhibition significantly reversed the HG-mediated upregulation of the PD-L1 protein (Fig. 3B). We found that inhibition of STAT3 and RAS signaling as well as *KRAS* si (Additional file 9: Fig. S9B) significantly inhibited *PD-L1* mRNA and protein levels (Fig. 3C, D, E), whereas PI3K signaling inhibition only slightly downregulated the PD-L1 protein (Additional file 9: Fig. S9C). Co-inhibition of STAT3 and RAS signaling led to a further decrease in *PD-L1* mRNA and protein levels (Fig. 3F, G).

To explore the reason for the downregulation of *PD-L1* mRNA, we examined whether high glucose affects the half-life of *PD-L1* mRNA. Following the inhibition of transcription with actinomycin D, high glucose treatment significantly prolonged the half-life of the *PD-L1* mRNA in PANC-1 and SW1990 cell lines. No effect on the half-life of the *PD-L1* mRNA was observed by knocking down STAT3, whereas inhibition of *KRAS* significantly reduced the half-life of the *PD-L1* mRNA (Fig. 3H).

These results indicate that high concentrations of glucose activated EGFR in pancreatic tumor cells, leading to upregulation of RAS signaling pathways and an increase in *PD-L1* mRNA levels via post-transcriptional mechanisms.

### RAS signaling pathway enhances stability of the *PD-L1* mRNA by downregulating PTRH1

**Fig. 4:**
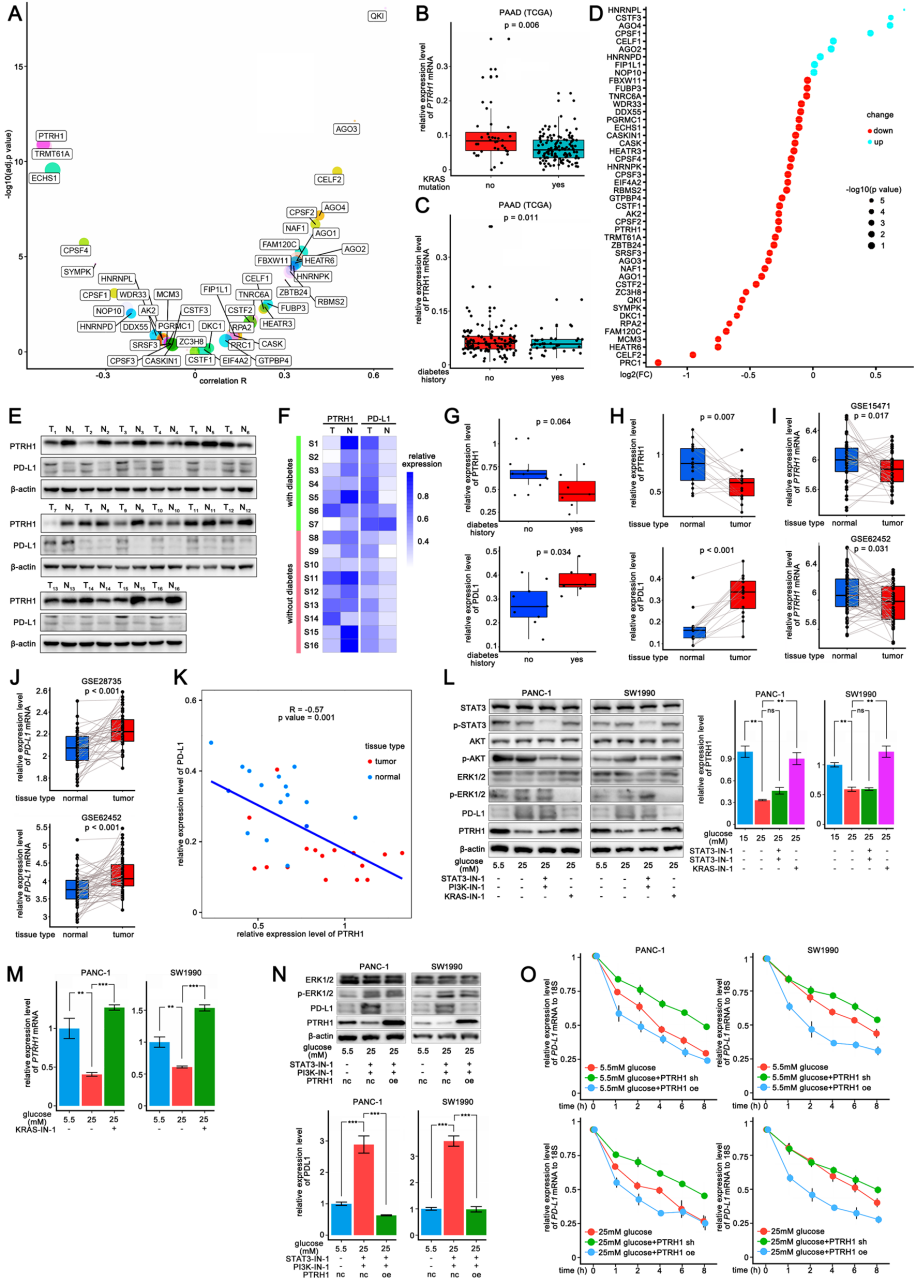
RAS signaling pathway enhance stability of *PD-L1* mRNA by down-regulating PTRH1. **A** Dot plot depicting the correlation between the mRNA level of RBPs and *PD-L1* in 178 PDAC samples from TCGA data. **B** Box plot depicting the differential level of *PTRH1* mRNA between PDAC samples with wild *KRAS* (n = 65) and mutant *KRAS* (n = 113) from TCGA data. **C** Box plot depicting the differential level of *PTRH1* mRNA between PDAC samples complicated with diabetes (n = 38) and those without diabetes (n = 108) from TCGA data. **D** Dot plot depicting the changes of the mRNA expression level of RBPs in tumor epithelial cells of PDAC samples complicated with diabetes (n = 10) compared to those without diabetes (n = 14) from single cell RNA-seq data (CRA001160). **E**–**F** Western blotting analysis **E** and Heatmap **F** of PTRH1 and PD-L1 expression in operative specimens of PDAC tissue (n = 16) and adjacent tissue (n = 16). **G** Box plot depicting the differential expression of PTRH1 and PD-L1 between PDAC samples complicated with diabetes (n = 7) and those without diabetes (n = 9). **H** Box plot depicting the differential expression of PTRH1 and PD-L1 between PDAC tissue (n = 16) and adjacent tissue (n = 16). **I** Box plot depicting the differential level of *PTRH1* mRNA between PDAC samples (n = 39) and adjacent samples (n = 39) from public data (GSE15471). **J** Box plot depicting the differential level of *PD-L1* mRNA between PDAC samples (n = 45) and adjacent samples (n = 45) from public data (GSE28735). **K** Correlation analysis of protein level of PTRH1 and PD-L1 in operative specimens (n = 32). **L** Western blotting analysis of PTRH1 and PD-L1 expression in PANC-1 and SW1990 cells following 24 h treatment with different inhibitors (STAT3-IN-1, KRAS-IN-1, PI3K-IN-1) in 25 mM sugar concentration medium. **M** qPCR analysis of mRNA level of *PTRH1* in PANC-1 and SW1990 cells following 24 h treatment with KRAS-IN-1 in 25 mM sugar concentration medium. **N** Western blotting analysis of PD-L1 expression in PANC-1 and SW1990 cells with overexpression of PTRH1 following 24 h treatment with combination of STAT3-IN-1 and PI3K-IN-1 in 25 mM sugar concentration medium. **O** qPCR analysis of *PD-L1* mRNA stability in PANC-1 (PTRH1 KD, NC, PTRH1 OE) and SW1990 cells (PTRH1 KD, NC, PTRH1 OE) after the addition of actinomycin D (10 μg/ml) in 15 mM or 25 mM sugar medium. *PDAC* pancreatic ductal adenocarcinoma, *TCGA* The Cancer Genome Atlas, *RBP* RNA binding protein, *OE* overexpression, *KD* knock down, *NC* negative control, *ns* non-significant. The graphs of Western blotting and qPCR show representative results from three independently repeated experiments. *p  < 0.05, **p  < 0.01, ***p  < 0.001

We analyzed the correlation of 1542 RBPs [25] and *PD-L1* mRNA level in PDAC samples from TCGA (n = 178), and selected top 100 RBPs that significantly negatively correlated to *PD-L1*. We predicted the probabilities of the interactions between these RBPs and the *PD-L1* 3′-UTR using the RPISeq website script (http://pridb.gdcb.iastate.edu/RPISeq/). In the RBPs that obtained high scores, PTRH1 was the only one that have been identified as potential regulatory factors affecting the stability of the *PD-L1* mRNA by previous study [9]. On the other hand, among those potential regulatory RBPs to *PD-L1* mRNA [9], PTRH1 showed the strongest negative relationship with PD-L1 expression (r = − 0.50, *p value* < 0.001) (Fig. 4A). In addition, *KRAS*-mutated PDAC samples (n = 113) presented lower *PTRH1* mRNA levels than wild-type *KRAS* samples (n = 65) from TCGA (Fig. 4B). In fact, in cancers with frequent *KRAS* mutations, including PDAC, lung adenocarcinoma (LAUD) and colon adenocarcinoma (COAD), *PTRH1* mRNA expression was significantly negatively correlated with *KRAS* mRNA levels (Additional file 10: Fig. S10).

The *PTRH1* mRNA level was found to be lower in PDAC samples from patients with diabetes from TCGA (n = 146) (Fig. 4C), and the same was true when comparing neoplastic epithelial cells in PDAC single-cell sequencing data (n = 24) (GSA: CRA001160) [26] (Fig. 4D). To examine the expression of PTRH1 in pancreatic cancer, we collected 16 cases of paired PDAC and adjacent tissue samples during surgery and identified the expression of PTRH1 and PD-L1 protein (Fig. 4E, F). According to our results, PDAC samples with diabetes (n = 7) had lower levels of PTRH1 (*p value* = 0.064) (Fig. 4G) and significantly more PD-L1 (*p value* = 0.034) (Fig. 4G) than those without diabetes (n = 9). In addition, the differential expression of PTRH1 and PD-L1 in PDAC and adjacent tissue was considerable (Fig. 4H), which corroborated the differential expression of *PTRH1* mRNA as well as *PD-L1* in PDAC and adjacent tissue in the public transcriptome sequencing datasets GSE15471 (n = 78), GSE62452 (n = 130), and GSE28735 (n = 90) (Fig. 4I, J). The restricted expression of PTRH1 in PDAC tissues can be attributed to the amplified oncogenic signaling including RAS. In a publicly available dataset containing 309 PDAC cases [27], samples expressing lower levels of PTRH1 enriched multiple oncogenic signaling pathways, such as the RAS, PI3K-Akt, and Wnt signaling pathways, whereas those samples expressing higher PTRH1 were enriched in some terms such as necroptosis and apoptosis (Additional file 11: Fig. S11). Among the samples collected (n = 32), the expression of PTRH1 was negatively correlated with the expression of PD-L1 (Spearman, r = − 0.57, *p value* = 0.001) (Fig. 4K). Taken together, these results suggest that PTRH1 expression may be suppressed by oncogenic signaling and high concentrations of blood glucose.

Therefore, we hypothesized that PTRH1 is a key RBP in the regulation of *PD-L1* mRNA stability via the RAS signaling pathway. As expected, high glucose levels suppressed PTRH1 protein expression in PANC-1 and SW1990 cell lines, and inhibition of STAT3 and Akt phosphorylation had no significant effect on PTRH1 expression, except inhibition of the RAS pathway, which restored PTRH1 expression (Fig. 4L and M). Moreover, overexpression of PTRH1 reversed PD-L1 protein expression in pancreatic tumor cells when cultured in 25 mM sugar medium under the combined inhibition of STAT3 and PI3K phosphorylation (Fig. 4N). We then knocked down PTRH1 in PANC-1 and SW1990 cell lines and observed a prolonged half-life of the *PD-L1* mRNA. Overexpression of PTRH1 significantly shortened the half-life of the *PD-L1* mRNA, and subsequent high-concentration glucose conditions were no longer able to prolong it (Fig. 4O).

### PTRH1 might inhibit PD-L1 expression by binding to the *PD-L1* mRNA 3′-UTR

**Fig. 5:**
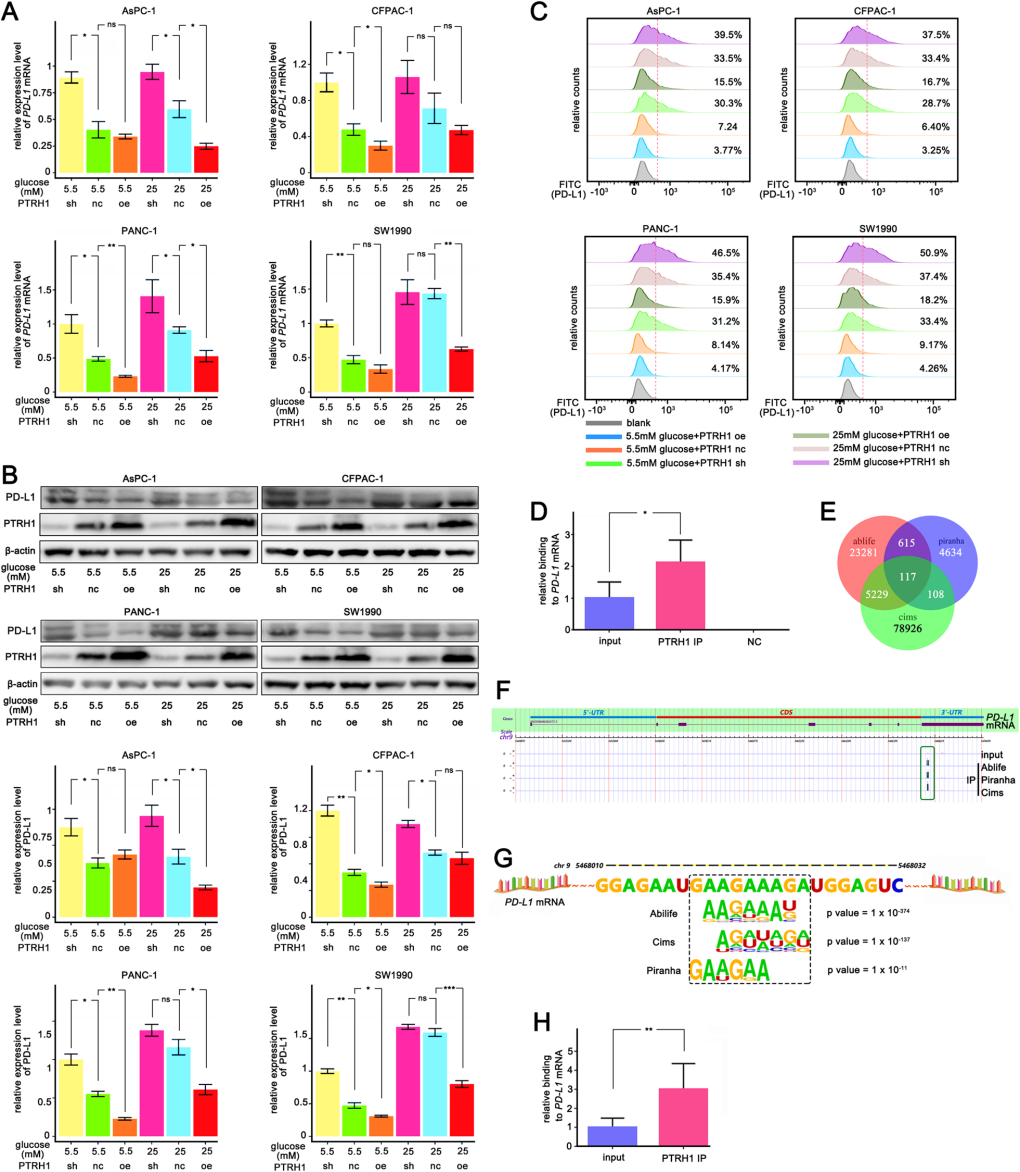
PTRH1 inhibits PD-L1 expression by binding *PD-L1* mRNA 3'-UTR **A-C** qPCR analysis **A** and Western blotting **B** and Flow cytometry analysis **C** of *PD-L1* expression in PANC-1, SW1990, AsPC-1, and CFPAC-1 following different treatment with overexpression or knocking down of PTRH1 in 5.5 mM or 25 mM sugar concentration medium. **D** qPCR analysis of the *PD-L1* mRNA content in RNAs obtained by pull-down of endogenous overexpressed PTRH1 protein in SW1990 cell lines. Anti-IgG antibody was used as a control. **E** Veen plot depicting overlapping binding peaks identified by three independent methods (Ablife, Piranha, and Cims) that PTRH1 may combine and interact to. RNA library was obtained by iRIP-seq experiment. **F** PTRH1 binding peaks on the *PD-L1* mRNA 3′-UTR in three analytic strategies (Ablife, Piranha, and Cims). **G** A preferred motif that PTRH1 binding to on the *PD-L1* mRNA 3′-UTR. **H** qPCR analysis of the binding of PTRH1 to *PD-L1* mRNA based on the cDNA library obtained from iRIP-seq experiment. *iRIP* Improved RNA Binding Protein Immunoprecipitation, *ns* non-significant. The graphs show representative results from three independently repeated experiments. *p  < 0.05, **p  < 0.01, ***p  < 0.001

We investigated the function of PTRH1 in regulating PD-L1 expression in pancreatic cancer cells. We knocked down and overexpressed PTRH1 in PANC-1, SW1990, AsPC-1, and CFPAC-1 cells which had shown PD-L1 upregulation in high glucose culturing. When cultured in 5.5 mM sugar medium, knockdown of PTRH1 upregulated the *PD-L1* mRNA and protein levels in all cell lines, and overexpression of PTRH1 significantly inhibited *PD-L1* mRNA and protein expression (Fig. 5A, B). For cells cultured with 25 mM sugar medium, overexpression of PTRH1 inhibited PD-L1 expression in PANC-1 and SW1990 cells when compared to the PTRH1 NC group (Fig. 5A, B).The results of the flow-cytometric analysis were similar to the Western blot finding (Fig. 5C).

Next, we tested whether PTRH1 regulates PD-L1 by binding to the *PD-L1* mRNA 3′-UTR to reduce the mRNA stability. We found that endogenous PTRH1 co-precipitated with the *PD-L1* mRNA in RNA immunoprecipitation (RNA-IP) assays in SW1990 cells (Fig. 5D). To determine the exact binding site, we used an improved RNA immunoprecipitation-sequence (iRIP-seq), and ultimately identified 117 genes with which PTRH1 may combine and interact (Fig. 5E, Additional file 16: Table S3). Putative binding peaks were observed in the *PD-L1* mRNA 3′-UTR in all three analytical strategies (Fig. 5F). Further statistical data analysis showed that the overlap peak on the *PD-L1* mRNA 3′-UTR was between approximately 5468000 and 5468050 on the chromosome 9 positive-strand, where a preferred motif, GAAGAAAGA, to which PTRH1 binds, is present (Fig. 5G). We detected the abundance of the *PD-L1* mRNA using the cDNA library obtained from iRIP-seq experiments, and found that compared with the input, the abundance of the IP group was significantly higher, which is consistent with previous results (Fig. 5H).

### PTRH1 overexpression rescues T cell function from high glucose-induced inhibition of pancreatic cancer cells

**Fig. 6:**
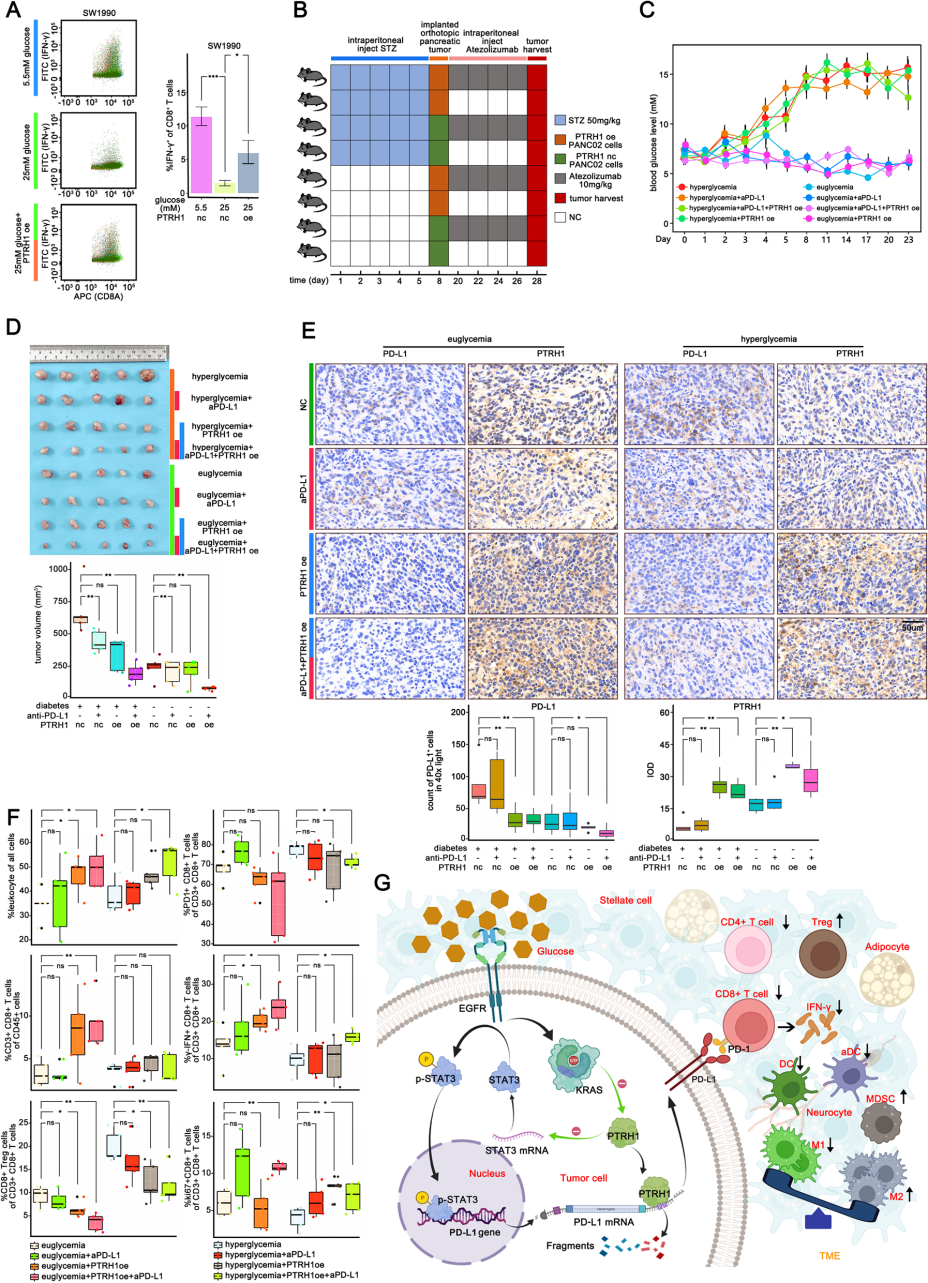
Overexpression of PTRH1 rescued T cell function from high glucose induced inhibiting effect of pancreatic cancer cells **A** Flow cytometry analysis of IFN-γ production of CD8^+^ T cells when cocultured with differentially treated SW1990 cells (5.5 mM concentration glucose, 25 mM concentration glucose, 25 mM concentration glucose + PTRH1 overexpression). **B** Experimental scheme and the details of experimental intervention in immune-competent C57BL/6 mice model (eight different treatment group with 5 mice per group). **C** The alterations in blood glucose levels after STZ treatment or fresh sodium citrate buffer as control in the mouse model. Blood was collected from mouse tails daily for the first 5 days after administration to measure blood glucose levels. Blood glucose was measured every 3 days thereafter until day 23. **D** Representative pictures and Box plot of the orthotopic tumors in immune-competent C57BL/6 mice in different groups. The pancreatic cancer cells were injected into the tail of the pancreas of mice and the tumors were harvested 3 weeks later. **E** Immunohistochemical staining to evaluate the distribution and level of PTRH1 and PD-L1 expression in the orthotopic tumors in immune-competent C57BL/6 mice in different groups. **F** Flow cytometry analysis of the proportion of infiltrated immune effectors (CD45^+^ cells, CD3^+^ T cells, Tregs, CD3^+^ CD8^+^ T cells, IFN-γ^+^ CD8^+^ T cells, PD-1^+^ CD8^+^ T cells, KI67^+^ CD8^+^ T cells) in the orthotopic tumors in immune-competent C57BL/6 mice in different groups. **G** Work model depicting the mechanism underlying the regulation on PD-L1 by high glucose and the changes of immune landscape in pancreatic TME. *DC* dendric cell, *MDSC* myeloid-derived suppressor cell, *ns* non-significant, *p  < 0.05, **p  < 0.01, ***p < 0.001

We co-cultured SW1990 cells subjected to various treatments with activated T cells in vitro. Our results showed that overexpression of PTRH1 in pancreatic cells weakened the immunosuppression induced by high glucose (Fig. 6A). Next, we demonstrated the potential of PTRH1 to improve anti-tumor immunological effects in the pancreatic TME. We established a diabetes model in C57 mice and implanted an orthotopic pancreatic tumor when the mice blood glucose reached the standard of diabetes (> 11.1 mmol/l) to describe the different immune landscapes of the pancreatic TME with different blood levels and PTRH1 expression (Fig. 6B, C). There were 8 different group with 5 mice per group. The experimental scheme and details of the intervention are shown in Fig. 6D. The average size of the tumors from control hyperglycemia group was larger than euglycemia group. Within the hyperglycemia mice, PTRH1 overexpression was more effective than anti-PD-L1 therapy in tumor suppression, and the combination of which exerted a substantial synergistic effect (Fig. 6D). Separately, overexpression of PTRH1 significantly reduced the growth of implanted tumors in hyperglycemia group (*p value* < 0.01). Interestingly, anti-PD-L1 treatment slightly shrank tumors when compared to the NC groups and demonstrated a synergistic effect with PTRH1 overexpression. In euglycemic mice, monotherapy failed to produce a desirable therapeutic effect. Overexpression of PTRH1 combined with anti-PD-L1 treatment showed a satisfactory treatment effect. IHC staining revealed significantly increased PD-L1 expression levels in wild-type pancreatic cancer cells in the hyperglycemia group compared to those in the euglycemia group (Fig. 6E), whereas PTRH1 overexpression significantly decreased PD-L1 signal intensity in pancreatic cancer cells in hyperglycemic mice.

The immunological effects of PTRH1 were further explored. According to our data (Fig. 6F, Additional file 12: Fig S12), PTRH1-overexpressing tumors contained more infiltrating immune cells than the negative control tumors. PD-L1 antibody treatment or overexpression of PTRH1 independently reduced the proportion of CD8^+^ Tregs in total CD8^+^ T cells, and a combination of both interventions further significantly reduced this distribution. In addition, only the combined treatment decreased the proportion of CD4^+^ Tregs among the total CD4^+^ T cells in hyperglycemic mice. All measures failed to change the proportion of CD4^+^ Tregs among CD4^+^ T cells in euglycemic mice.

Although IFN-γ^+^CD8^+^ or Ki67^+^CD8^+^ T cells were relatively scarce, the combination of PTRH1 overexpression and anti-PD-L1 treatment successfully elevated the relative abundance of these two cell populations in both euglycemic and hyperglycemic mice, while the euglycemic mice tended to have more IFN-γ^+^CD8^+^ T cells than the hyperglycemic ones (Fig. 6F).

Tumors in hyperglycemic mice contained more macrophages and MDSCs but less DCs compared to those in euglycemic mice (Additional file 12: Fig. S12). Interestingly, the M2-to-M1 macrophage ratio was significantly higher in hyperglycemic mice, while neither PTRH1 overexpression or anti-PD-L1 treatment altered this ratio. Notably, both total and activated DCs were increased by PTRH1 overexpression and/or anti-PD-L1 treatment in hyperglycemic mice. The changes of immune landscape in pancreatic TME driven by high glucose is shown in Fig. 6G.

## Discussion

Diabetes is a growing global public health issue contributing to morbidity, disability, and premature mortality [28]. In the absence of obvious clinical symptoms, the toxic effects of glucose can cause pathological and functional changes in different tissues and organs [29]. Diabetic subjects are more susceptible to infection [30], and the outcome of infection treatment in patients who suffer from diabetes tends to be poor [31]. The immunological mechanism of susceptibility of diabetics to infections may be due to multiple immunosuppressive factors including suppression of cytokine production, defect in leukocyte recruitment, and neutrophil dysfunction [32]. Besides, both Type 1 and Type 2 diabetes mellitus have been increasingly recognized as risk factors for the development of various cancers, including PDAC [33, 34]. However, the impact of diabetes on the immune landscape pattern in the pancreatic TME remain elusive.

The relationship between cellular glucose metabolism and PD-L1 expression has been complicated. High glucose activates EGFR and promotes hexokinase (HK) 2 dissociation from mitochondria and its subsequent binding and phosphorylation of I κbα at T291, which finally lead to NF-κB activation-dependent transcriptional upregulation of PD-L1 expression [35]. The downstream signaling pathways of EGFR including JAK/STAT3 [36], RAS/ERK [23] and PI3K/Akt [37] have been reported to be related to the up-regulation of PD-L1 expression. Our previous study have demonstrated that high glucose can enhance glycolysis through upregulating Bmi1-UPF1-HK2 pathway and inhibit anti-tumor effectors in pancreatic TME [38]. In addition, the glycolysis intensity was significantly correlated with the expression of PD-L1 in CD86^+^ macrophages in liver cancer and the mechanism involves HIF-1α-dependent elevation of PKM2 [39]. Lactic acid produced by glycolysis is also a potent stimulus to PD-L1 of cancer cells [40] or macrophages [41]. Tumor exosomes induce glycolytic metabolic reprogramming by stimulating TLR2 receptors in tumor-associated macrophages and up-regulate PD-L1 expression in a NFKB dependent manner [41]. At the post-translational level, high glucose inhibits phosphorylation of the energy baroreceptor AMPK, which induces its targeted lysosome degradation by phosphorylation of PD-L1 [42]. The regulation of glucose on PD-L1 expression is related to the concentration of glucose and the type of cancer cells. It has been reported that low concentration glucose (500 mg/l) can up-regulate PD-L1 expression by over-activating the downstream ERK pathway through up-regulating EGFR expression in renal cancer cells [43]. In triple-negative breast cancer cell lines, low glucose also causes upregulation of PD-L1, and this upregulation can be eliminated by 2-DG or metformin. Interestingly, PD-L1 also affects the glucose metabolism of cells [44]. The blocking of PD-L1 can inhibit the glycolysis of cancer cells by inhibiting the activity of mTOR, thereby relieving the pressure of glucose deficiency in the tumor microenvironment and allowing the glycolysis and IFN-γ production of infiltrated T cells [45]. Retinoic Acid-Related Orphan Receptor C regulates proliferation, glycolysis, and chemoresistance via the PD-L1/ITGB6/STAT3 signaling axis in bladder cancer [46]. It is worth noting that a very small number of patients treated by ICI will develop fatal type I diabetes. It is especially necessary to monitor blood sugar for pancreatic cancer patients complicated with diabetes to cope with adverse events [47, 48].

Our data suggest that high glucose levels worsen the immunosuppressive pattern of the pancreatic cancer microenvironment, with an increased level of macrophage infiltration and a decreased proportion of infiltrating T cells accompanied by reduced functions. High glucose significantly increased *PD-L1* mRNA stability by stimulating EGFR to activate downstream RAS signaling. It has been suggested that RAS signaling is unlikely to regulate PD-L1 expression via a transcriptional mechanism because it fails to induce expression of PD-L1 promoter-controlled reporter constructs in H358 cells [23]. PTRH1 was speculated to be the connecting node based on bioinformatics data, which was subsequently proven experimentally. PTRH1 is a 214 amino acid protein that belongs to the PTH family. The PTRH1 protein is believed to be involved in RNA splicing, silencing and metabolism.

Targeting programmed death 1 ligand 1 (PD-L1) or its receptor PD-1 (programmed cell death 1) has promising curative effects in several tumors with frequent RAS mutations such as non-small cell lung cancer (NSCLC) [49] and mismatch-repair-deficient colorectal cancer [50]. The benefits of nivolumab therapy in patients with NSCLC are related to tumor *KRAS* mutations [51]. Despite the presence of *KRAS* mutations in most PDAC cases [52], currently available PD-L1/PD-1 immunotherapies did not achieve satisfactory efficacy [53]. Resistance to anti-PD-1/PD-L1 immunotherapy develops in PDAC patients but the underlying mechanisms remain elusive. The low sensitivity of pancreatic cancer to PD-1/PD-L1 blockers contrasts the weak role of PD-L1 in the pancreatic TME, in which a highly dense tissue barrier might make exogenous intervention difficult to inhibit PD-L1 function [54]. Studies have shown that the efficacy of anti-PD-1/PD-L1 therapy is positively correlated with PD-L1 expression in cancer [55, 56]. However, there are controversial reports regarding the PD-L1 expression levels in pancreatic cancers. Some studies have reported that human pancreatic cancers express limited PD-L1 levels [49, 57], whereas others have shown that many PD-L1 proteins exist human pancreatic cancers, and nine of 10 pancreatic cancer cell lines are positive for PD-L1 [58]. There is no consensus on the threshold to determine whether a tumor is PD-L1-positive or PD-L1-negative, which is a challenge for comparisons between studies. Whether T cells in pancreatic cancer are inhibited by PD-L1 should be determined, which is the basis of anti-PD-L1/PD-1 therapy. On one hand, published single-cell data show significantly upregulated mRNA levels of multiple immune checkpoints, and especially PD-1, among pancreatic cytotoxic T cells compared to that in peripheral cytotoxic T cells (Additional file 13: Fig. S13). On the other hand, the expression of PD-L1 was increased in pancreatic cancer compared with that in adjacent tissues, and it was further increased under high glucose conditions according to our data. Studies have shown that PD-L1 expression is correlated with low tumor-infiltrating lymphocyte levels and poor prognosis [8, 59, 60].

In mice with complete immunity, PTRH1 overexpression inhibited tumor growth and significantly increased the infiltration and function of T cell. Significantly enhanced efficacy was observed in combination with anti-PD-L1. It should be noted that anti-PD-L1 may also directly suppress tumor growth by disrupting mTOR activity and glycolysis [45]. Anti-PD-1 therapy is known to stimulate PD-L1 expression in cancer cells, and subsequently induce resistance to Fas or staurosporine-mediated apoptosis, and protect them from the cytotoxic effects of type I and II interferons or the cytotoxic T lymphocyte (CTL)-mediated lysis [61, 62]. These observations may explain in part the synergistic effect of downregulation of PD-L1 in combination with anti-PD-1/PD-L1 therapy. Therefore, although pancreatic cancer has yet to show response to anti-PD-1/PD-L1 therapy, it would still be beneficial to understand the mechanism underlying the regulation of PD-L1 expression.

Siglec-15 exhibits a comparable domain composition and high homology to PD-L1, suggesting a potential synergistic effect in suppressing both T cell proliferation and activation [63]. The expression of Siglec-15 and PD-L1 has been reported to be mutually exclusive in several types of cancer, suggesting that targeting Siglec-15 may provide an alternative therapeutic strategy for cancers unresponsive to anti-PD-L1/PD-1 immunotherapy [64, 65]. A study utilized immunohistochemical staining to identify the expression of Siglec-15 and PD-L1 in 291 pancreatic adenocarcinoma tissue specimens, revealing a certain complementarity between the two in pancreatic cancer cells [66]. Positive Siglec-15 expression on pancreatic cancer cells was associated with improved progression-free survival (PFS) and disease-specific survival (DSS) [66], while PD-L1 exhibited an inverse correlation [67]. Notably, a separate report indicates that tumor-associated macrophages expressing Siglec-15 in the microenvironment of pancreatic cancer exhibit a distinct immunosuppressive phenotype that is sustained by SYK/MAPK activation associated with Siglec-15 [68]. In our study, high glucose significantly skewed tumor-associated macrophages in the tumor microenvironment towards an M2 phenotype. Glucose is a potent activator of the MAPK pathway and may also contribute to the maintenance of M2-like polarization in tumor-associated macrophages. Siglec-15 may enhance cancer cell invasion by promoting EGFR protein stability, as demonstrated in thyroid cancer [69]. Our findings indicate that high glucose levels activate EGFR and increase the stability of *PD-L1* mRNA, resulting in up-regulated expression of PD-L1 in pancreatic cancer cells. Therefore, it would be intriguing and worthwhile to investigate the expression of PD-L1 and Siglec-15 in various cell types and their reciprocal regulatory roles within the pancreatic cancer microenvironment under hyperglycemic conditions.

The study had a few limitations. Inferred RBPs that regulate the *PD-L1* mRNA were screened mainly through a literature search and biological information, instead of specific experiments. Further clinical studies are necessary to elucidate the association between PTRH1 expression and diabetes. The mechanism of KRAS regulating PTRH1 which can be an attractive point that was unclear still.

## Conclusion

we confirmed that PTRH1, as an RBP, plays a key role in PD-L1 regulation by high glucose and is closely related to anti-tumor immunity in the pancreatic cancer microenvironment.

## Supplementary Information

**Additional file 1: Figure S1 **iRIP-seq workflow.

**Additional file 2: Figure S2 **Pictures showing the orthotopic mouse pancreatic cancer models and the harvest of the implanted orthotopic pancreatic tumors of mice.

**Additional file 3: Figure S3 **The gating strategy of flow cytometry analysis in identifying infiltrating immune effectors in orthotopic pancreatic tumors of mice.

**Additional file 4: Figure S4** Single cell RNA-seq data processing of CD8^+^ T cells. **A** Processing of identification of CD8^+^ T cells. **B** Violin plot depicting the feature and count of genes as well as the percentage of mitochondrial genes. **C** Correlation analysis of feature genes to detect sequencing depth. **D** Variogram depicting variable feature genes among cells. **E** Principal Component Analysis (PCA) of cells. **F** Dot plot depicting feature genes of each Principal Component. **G** Heatmap depicting variable feature genes of PCA. **H** JackStraw Plot of PC1 to 20. **I** Dot plot depicting expression of markers of T cell subsets of clusters. Single cell RNA-seq data was downloaded from GSA: CRA001160.

**Additional file 5: Figure S5 **Single cell RNA-seq data processing of epithelial cells.** A** Processing of identification of epithelial cells. **B **Violin plot depicting the feature and count of genes as well as the percentage of mitochondrial genes. **C** Correlation analysis of feature genes to detect sequencing depth. **D** Variogram depicting variable feature genes among cells. **E** Principal Component Analysis (PCA) of cells. **F** Dot plot depicting feature genes of each Principal Component. **G** Heatmap depicting variable feature genes of PCA. **H** JackStraw Plot of PC1 to 20. **I** Dot plot depicting expression of markers of T cell subsets of clusters; Bar plot depicting the clusters existing in PDAC tissue and normal tissue. Single cell RNA-seq data was downloaded from GSA: CRA001160.

**Additional file 6: Figure S6 **proportion of immune subsets in in PDAC samples. **A **Stacked bar plot depicting proportion of cells in PDAC samples. **B **Violin plot depicting the comparison of cell proportions between PDAC samples complicated with diabetes and those without diabetes.

**Additional file 7: Figure S7 **Flow cytometry analysis of IFN-γ production of CD8^+^ T cells cocultured with pancreatic cancer cells that pretreated with 25 mM or 5.5 mM glucose.

**Additional file 8: Figure S8 **Flow cytometry analysis of IFN-γ production of CD8^+^ T cells under high concentrations of glucose or PD-L1 antibodies treatment individually

**Additional file 9: Figure S9 A** Western blotting analysis of PD-L1 expression in PANC-1 and SW1990 cells following 12 h treatment with AICAR in 5.5 mM glucose medium. **B** Western blotting analysis of PD-L1 expression in PANC-1 and SW1990 cells following 72 h treatment with KRAS or STAT3 si in 25 mM glucose medium. **C** Western blotting analysis of PD-L1 expression in PANC-1 and SW1990 cells following 48 h treatment with PI3K-IN-1 in 25 mM glucose medium. The graphs show representative results from three independently repeated experiments. *: *p value*< 0.05, **: *p value*< 0.01, ***: *p value*< 0.001

**Additional file 10: Figure S10 **Correlation analysis of mRNA level of *PTRH1* and *KRAS* in pancreatic adenocarcinoma (PAAD), lung adenocarcinoma (LAUD) and colon adenocarcinoma (COAD) samples from TCGA.

**Additional file 11: Figure S11 **KEGG pathway enrichment analysis based on differential genes between PDAC samples expressing higher *PTRH1* and those expressing lower *PTRH1* from E-MTAB-6134 dataset.

**Additional file 12: Figure S12 **Flow cytometry analysis of the infiltration of immune effectors (CD4^+^ T cells, MDSCs, macrophages and DCs) in the orthotopic tumors in immune-competent C57BL/6 mice in different groups.

**Additional file 13: Figure S13 **Immune checkpoints expression on CD8^+^ T cells in PDAC TME and peripheral blood. **A** Processing of identification of CD8^+^ T cells. **B** scatter plot depicting the differential level of several immune checkpoints on CD8^+^ T cells in PDAC TME and peripheral blood.

**Additional file14: Table S1 **Details for resource used.

**Additional file 15: Table S2 **Sheet1: Differential expression genes of CD8^+^ T cells in PDAC samples with diabetes and those without diabetes. Sheet2: Differential expression genes of epithelial tumor cells in PDAC samples with diabetes and those without diabetes

**Additional file 16: Table S3 **117 overlapping genes that PTRH1 may combine and interact with.

## Data Availability

The data analyzed in this study were obtained from TCGA; Gene Expression Omnibus (GEO): GSE155698 [70], GSE15471 [71], GSE62452 [72], and GSE28735 [73]; Genome Sequence Archive (GSA): CRA001160 [26]; and ArrayExpress (AE): E-MTAB-6134 [27].

## Abbreviations

PD-L1: Programmed death 1 ligand 1
PD-1: Programmed cell death 1
TME: Tumor microenvironment
PTRH1: Peptidyl-tRNA hydrolase 1 homolog
iRIP-seq: Improved RNA Binding Protein Immunoprecipitation sequence
RIP: RNA Binding Protein Immunoprecipitation
EGF: Epidermal growth factor
EGFR: Expression and transactivates EGF receptor
UTRs: Untranslated regions
RBPs: RNA binding proteins
ChIP: Chromatin immunoprecipitation assay
STZ: Streptozocin
DMEM: Dulbecco’s Modified Eagle Medium
PBMCs: Peripheral blood mononuclear cells
MDSCs: Myeloid-derived suppressive cells
AICAR: Aminoimidazole-4-carboxamide1-β-d-ribofuranoside
DEG: Differential expressed genes
DCs: Dendritic cells
