# Supplementary figures and images for: High glucose-upregulated PD-L1 expression through RAS signaling-driven downregulation of PTRH1 leads to suppression of T cell cytotoxic function in tumor environment
Source: J Transl Med. 2023 Jul 11;21:461. doi: 10.1186/s12967-023-04302-4 (PMC10337161; doi:10.1186/s12967-023-04302-4)

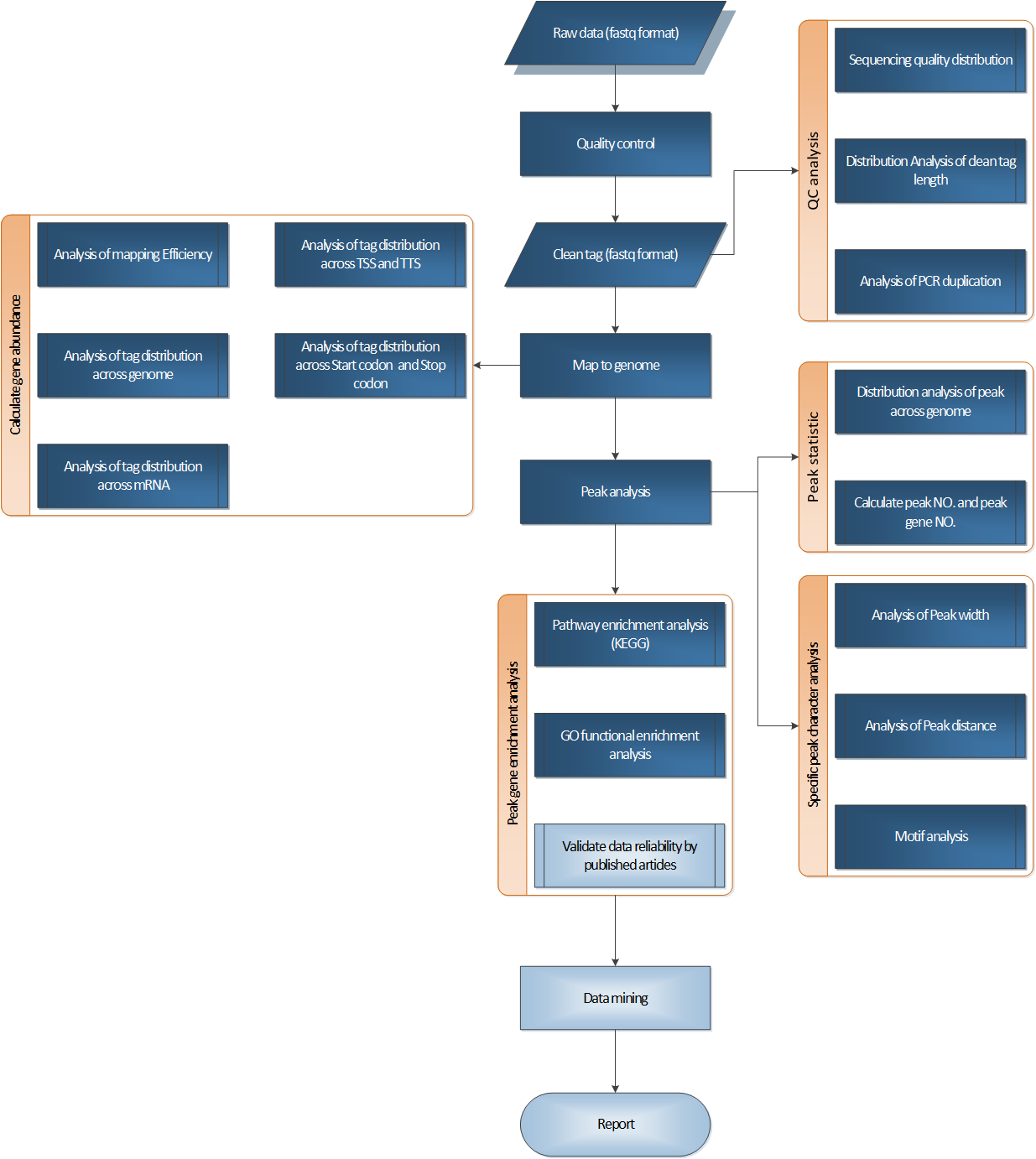

Supplement: Supplementary file 1 — Additional file 1: Figure S1 iRIP-seq workflow. [file 12967_2023_4302_MOESM1_ESM.png]

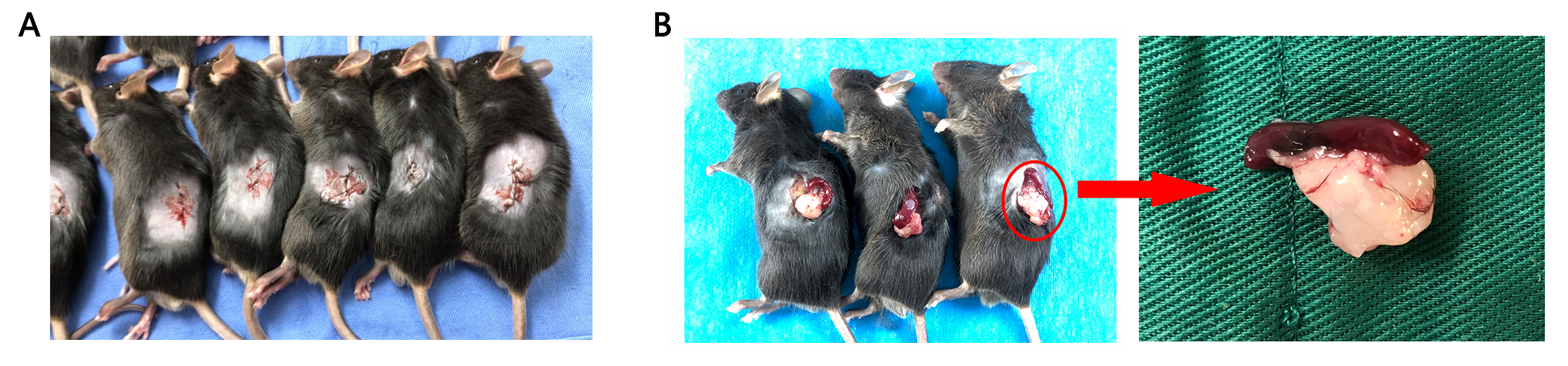

Supplement: Supplementary file 2 — Additional file 2: Figure S2 Pictures showing the orthotopic mouse pancreatic cancer models and the harvest of the implanted orthotopic pancreatic tumors of mice. [file 12967_2023_4302_MOESM2_ESM.tif]

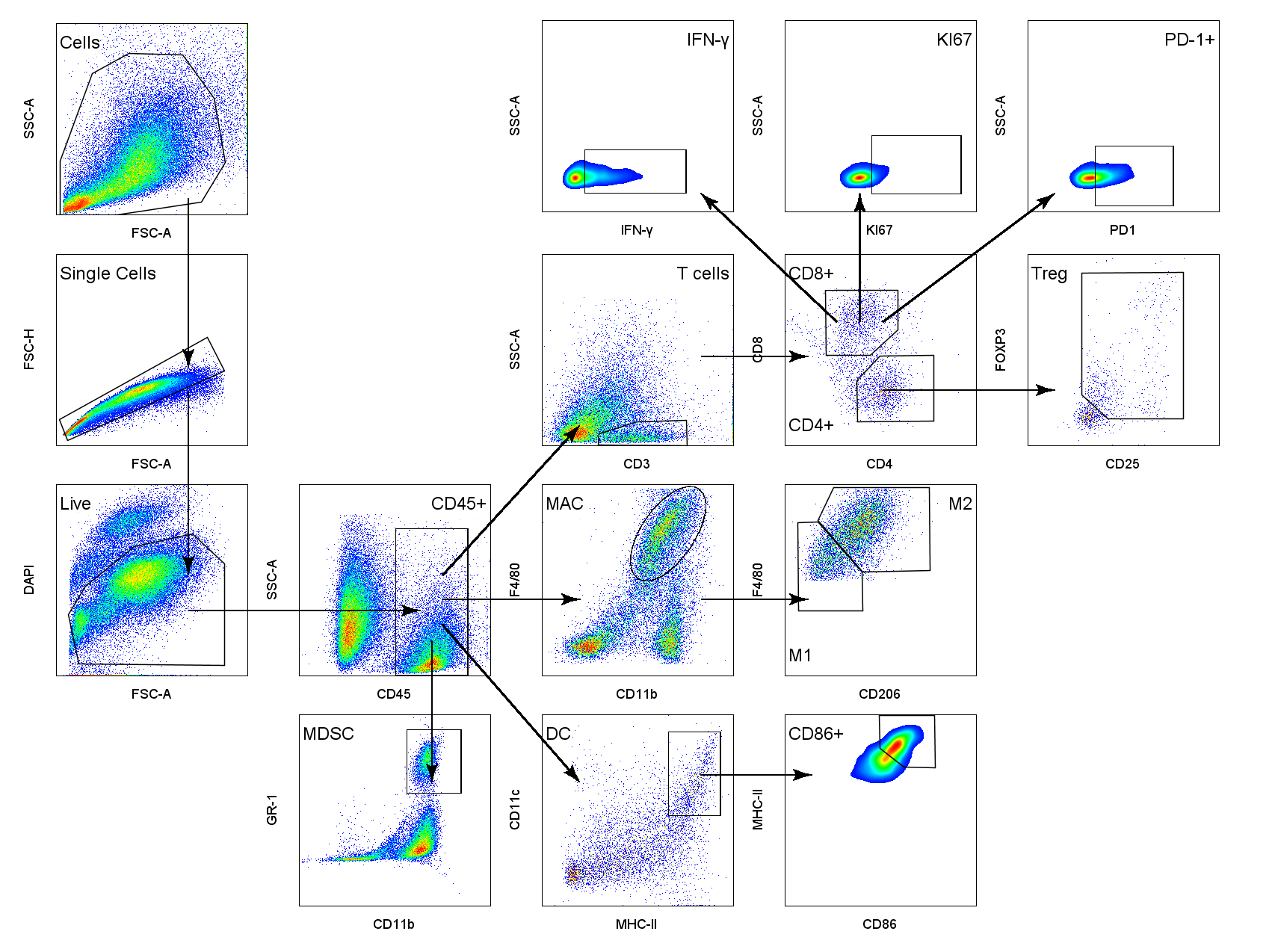

Supplement: Supplementary file 3 — Additional file 3: Figure S3 The gating strategy of flow cytometry analysis in identifying infiltrating immune effectors in orthotopic pancreatic tumors of mice. [file 12967_2023_4302_MOESM3_ESM.tiff]

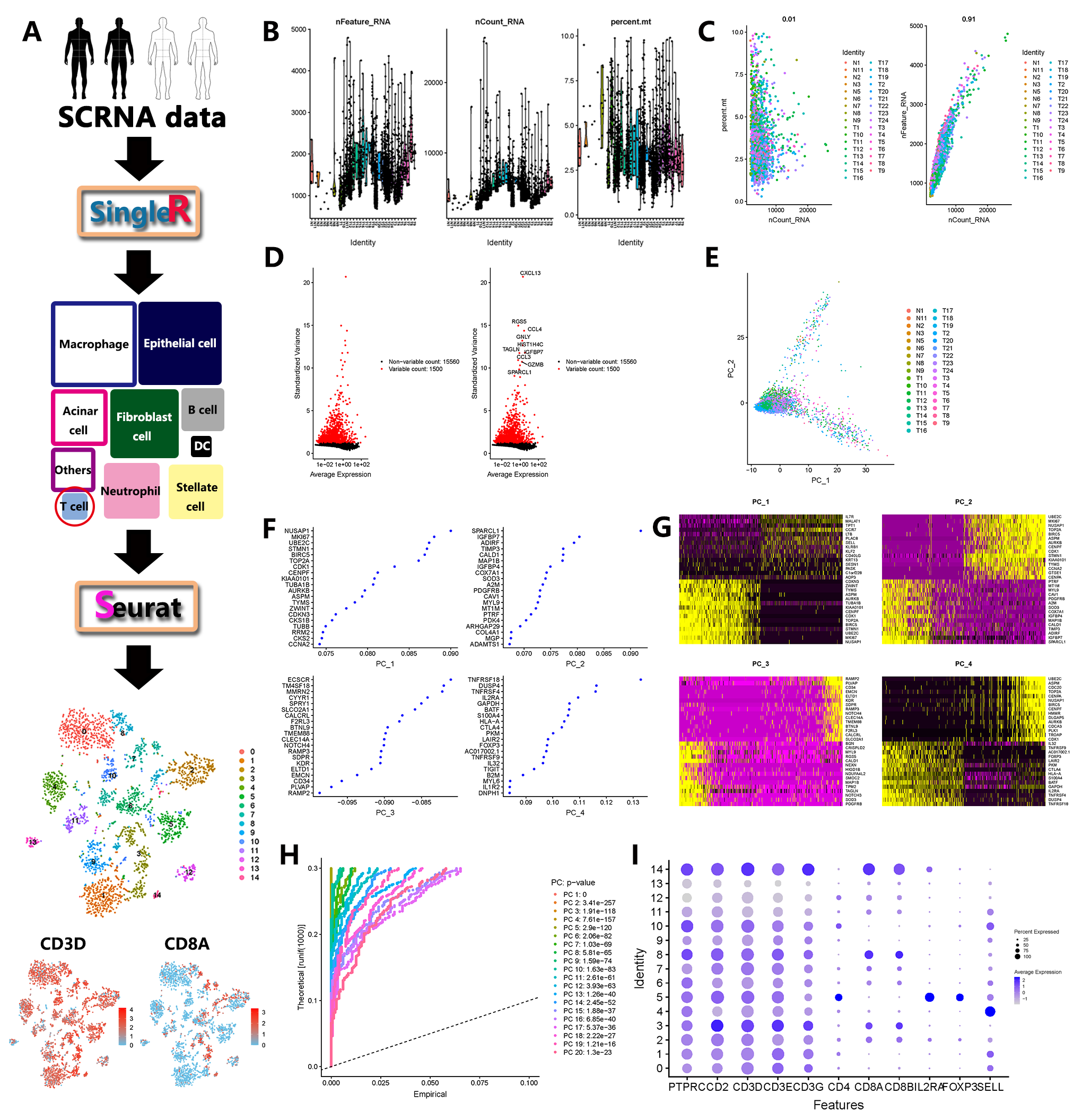

Supplement: Supplementary file 4 — Additional file 4: Figure S4 Single cell RNA-seq data processing of CD8+ T cells. A Processing of identification of CD8+ T cells. B Violin plot depicting the feature and count of genes as well as the percentage of mitochondrial genes. C Correlation analysis of feature genes to detect sequencing depth. D Variogram depicting variable feature genes among cells. E Principal Component Analysis (PCA) of cells. F Dot plot depicting feature genes of each Principal Component. G Heatmap depicting variable feature genes of PCA. H JackStraw Plot of PC1 to 20. I Dot plot depicting expression of markers of T cell subsets of clusters. Single cell RNA-seq data was downloaded from GSA: CRA001160. [file 12967_2023_4302_MOESM4_ESM.tif]

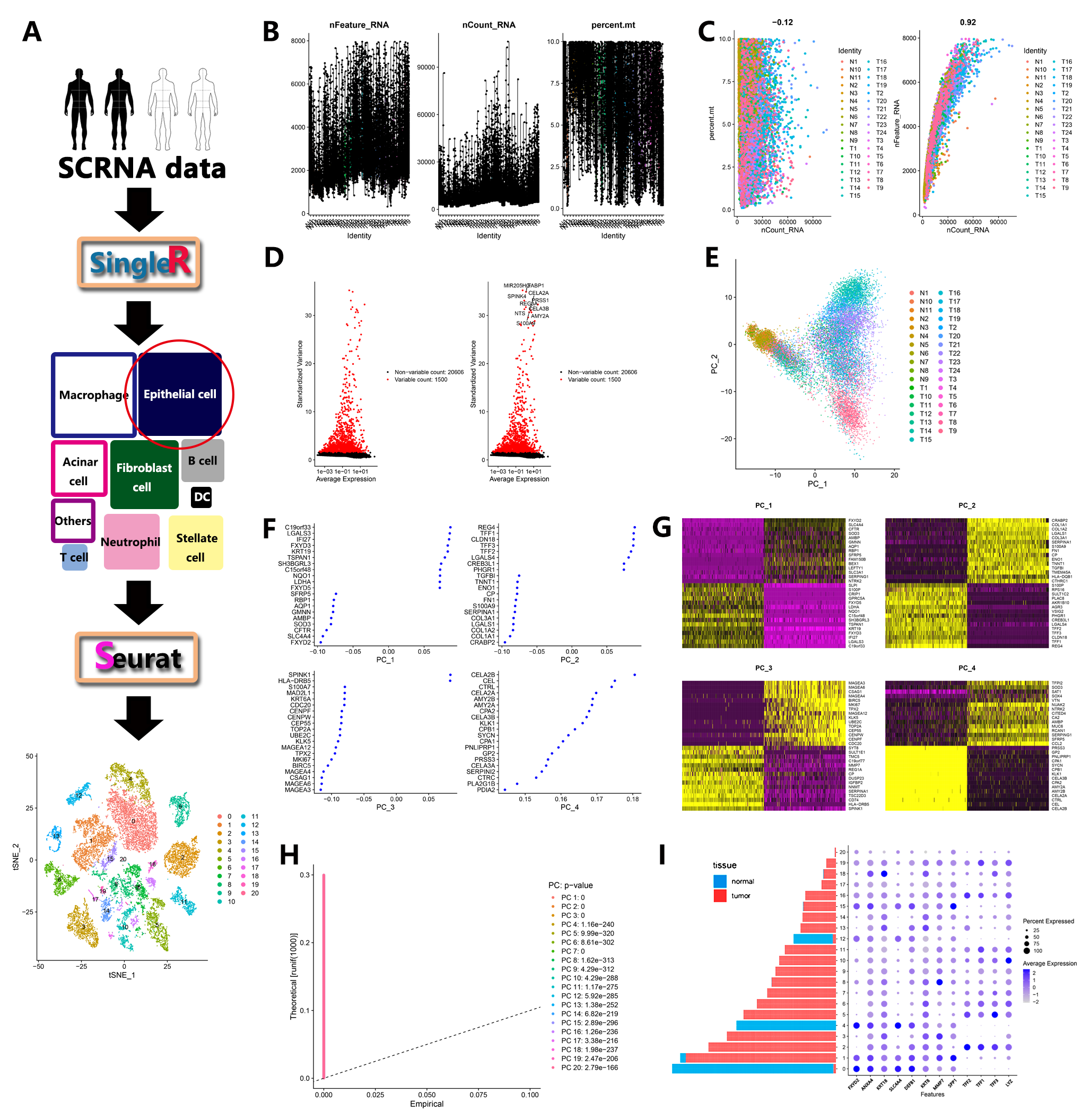

Supplement: Supplementary file 5 — Additional file 5: Figure S5 Single cell RNA-seq data processing of epithelial cells. A Processing of identification of epithelial cells. B Violin plot depicting the feature and count of genes as well as the percentage of mitochondrial genes. C Correlation analysis of feature genes to detect sequencing depth. D Variogram depicting variable feature genes among cells. E Principal Component Analysis (PCA) of cells. F Dot plot depicting feature genes of each Principal Component. G Heatmap depicting variable feature genes of PCA. H JackStraw Plot of PC1 to 20. I Dot plot depicting expression of markers of T cell subsets of clusters; Bar plot depicting the clusters existing in PDAC tissue and normal tissue. Single cell RNA-seq data was downloaded from GSA: CRA001160. [file 12967_2023_4302_MOESM5_ESM.tif]

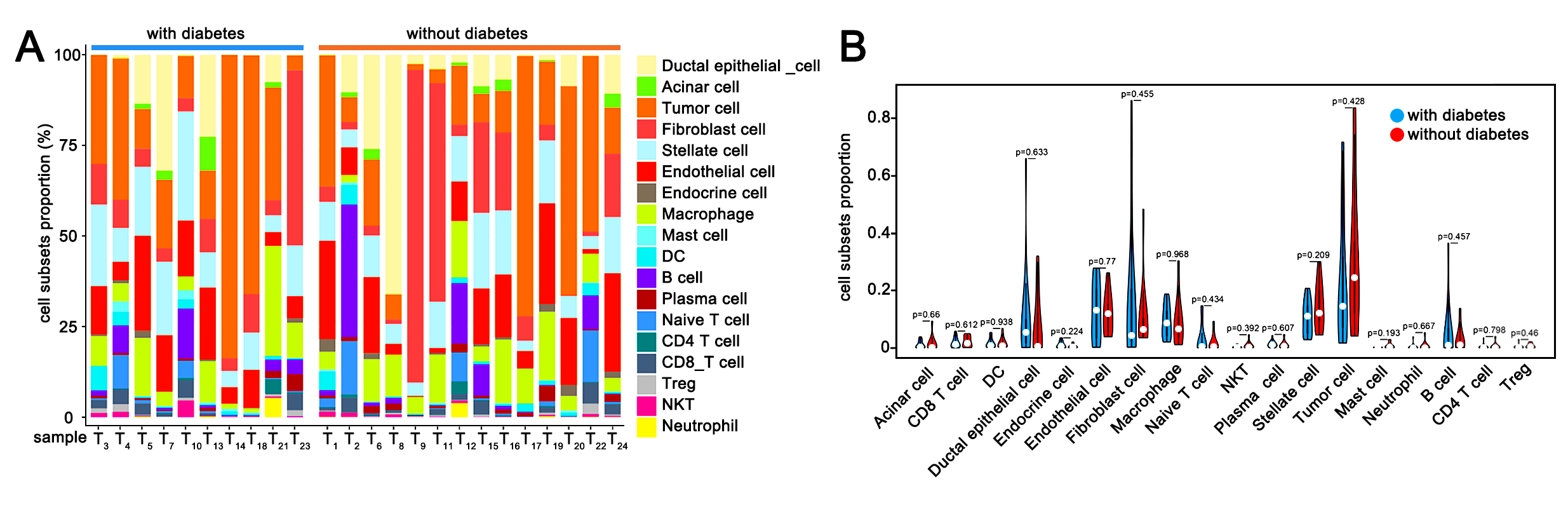

Supplement: Supplementary file 6 — Additional file 6: Figure S6 proportion of immune subsets in in PDAC samples. A Stacked bar plot depicting proportion of cells in PDAC samples. B Violin plot depicting the comparison of cell proportions between PDAC samples complicated with diabetes and those without diabetes. [file 12967_2023_4302_MOESM6_ESM.tif]

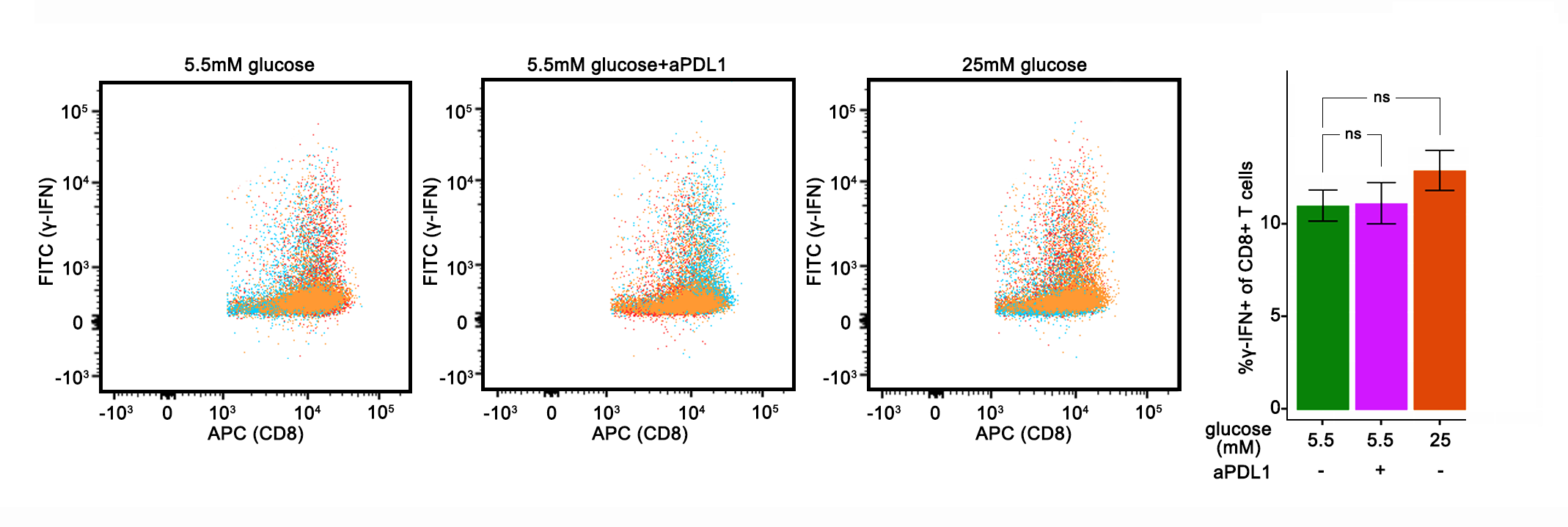

Supplement: Supplementary file 7 — Additional file 7: Figure S7 Flow cytometry analysis of IFN-γ production of CD8+ T cells cocultured with pancreatic cancer cells that pretreated with 25 mM or 5.5 mM glucose. [file 12967_2023_4302_MOESM7_ESM.tif]

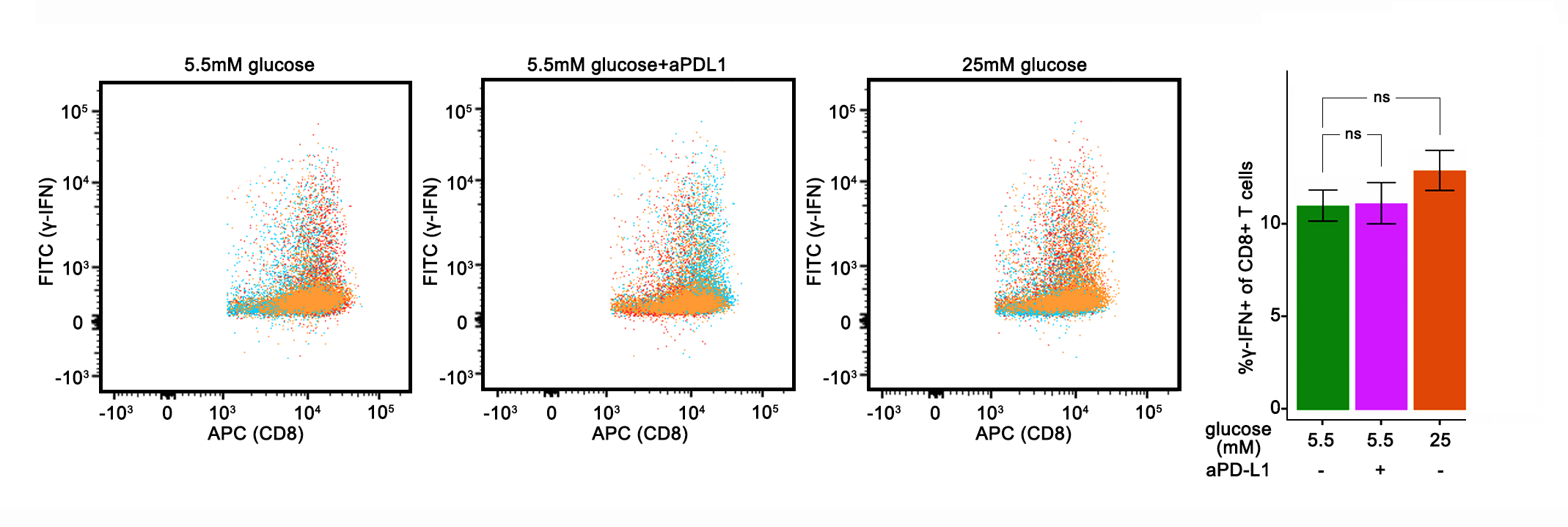

Supplement: Supplementary file 8 — Additional file 8: Figure S8 Flow cytometry analysis of IFN-γ production of CD8+ T cells under high concentrations of glucose or PD-L1 antibodies treatment individually [file 12967_2023_4302_MOESM8_ESM.tif]

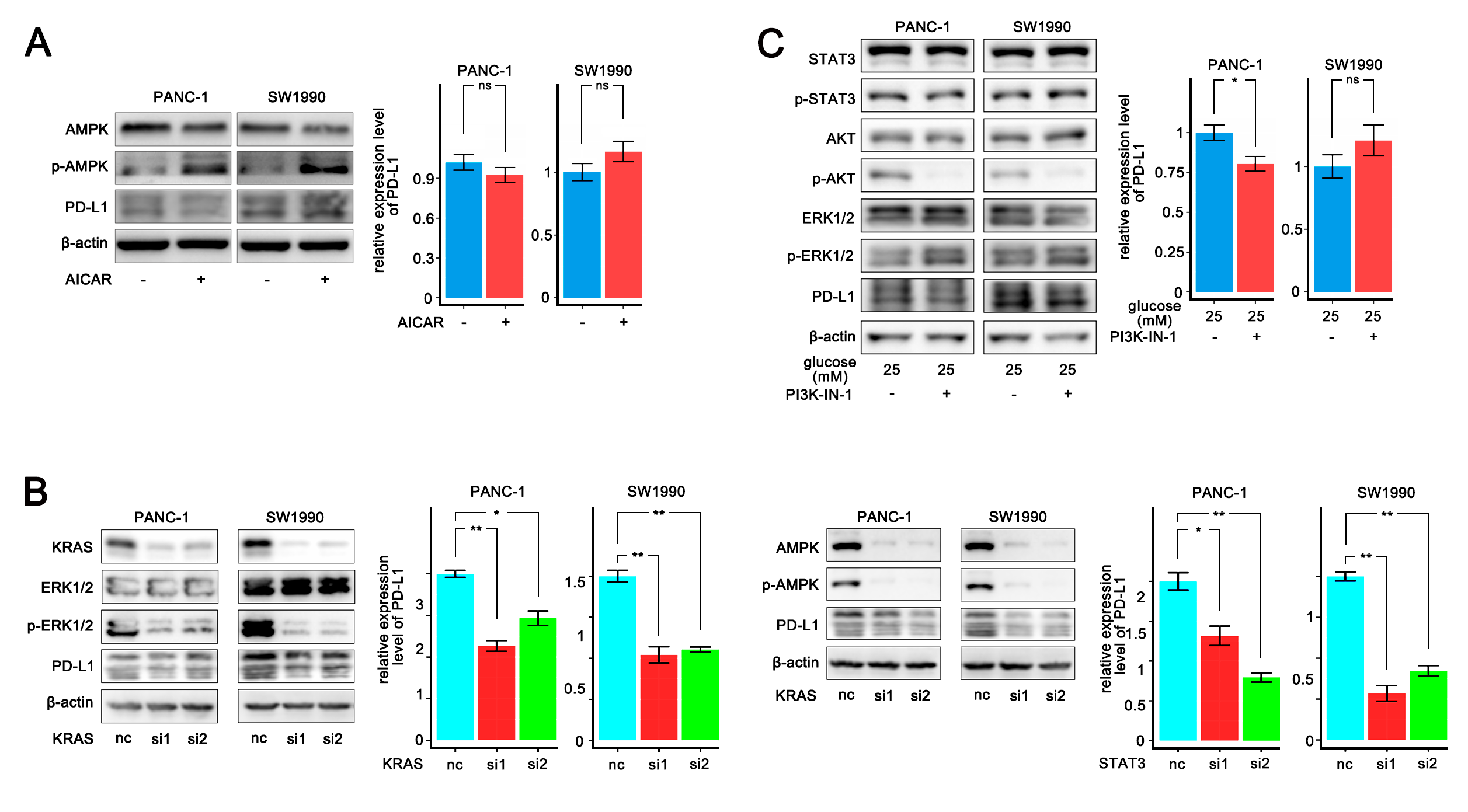

Supplement: Supplementary file 9 — Additional file 9: Figure S9 A Western blotting analysis of PD-L1 expression in PANC-1 and SW1990 cells following 12 h treatment with AICAR in 5.5 mM glucose medium. B Western blotting analysis of PD-L1 expression in PANC-1 and SW1990 cells following 72 h treatment with KRAS or STAT3 si in 25 mM glucose medium. C Western blotting analysis of PD-L1 expression in PANC-1 and SW1990 cells following 48 h treatment with PI3K-IN-1 in 25 mM glucose medium. The graphs show representative results from three independently repeated experiments. *: p value< 0.05, **: p value< 0.01, ***: p value< 0.001 [file 12967_2023_4302_MOESM9_ESM.tif]

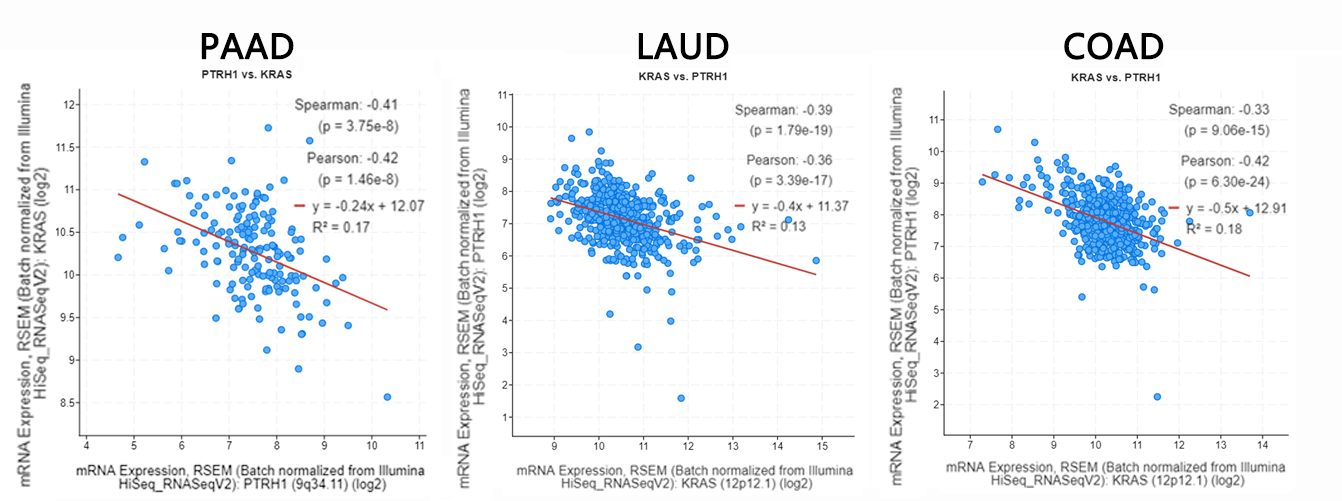

Supplement: Supplementary file 10 — Additional file 10: Figure S10 Correlation analysis of mRNA level of PTRH1 and KRAS in pancreatic adenocarcinoma (PAAD), lung adenocarcinoma (LAUD) and colon adenocarcinoma (COAD) samples from TCGA. [file 12967_2023_4302_MOESM10_ESM.tif]

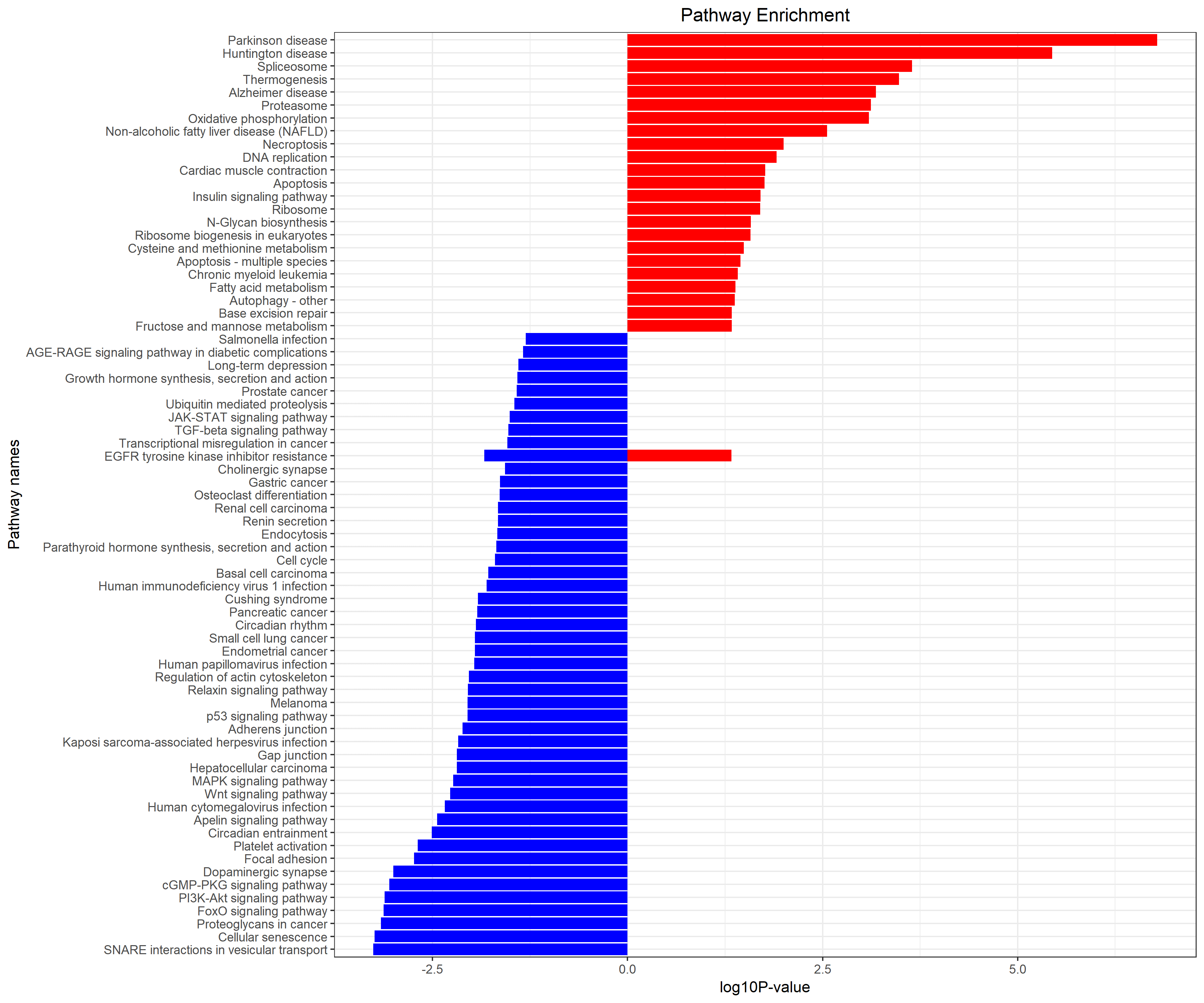

Supplement: Supplementary file 11 — Additional file 11: Figure S11 KEGG pathway enrichment analysis based on differential genes between PDAC samples expressing higher PTRH1 and those expressing lower PTRH1 from E-MTAB-6134 dataset. [file 12967_2023_4302_MOESM11_ESM.png]

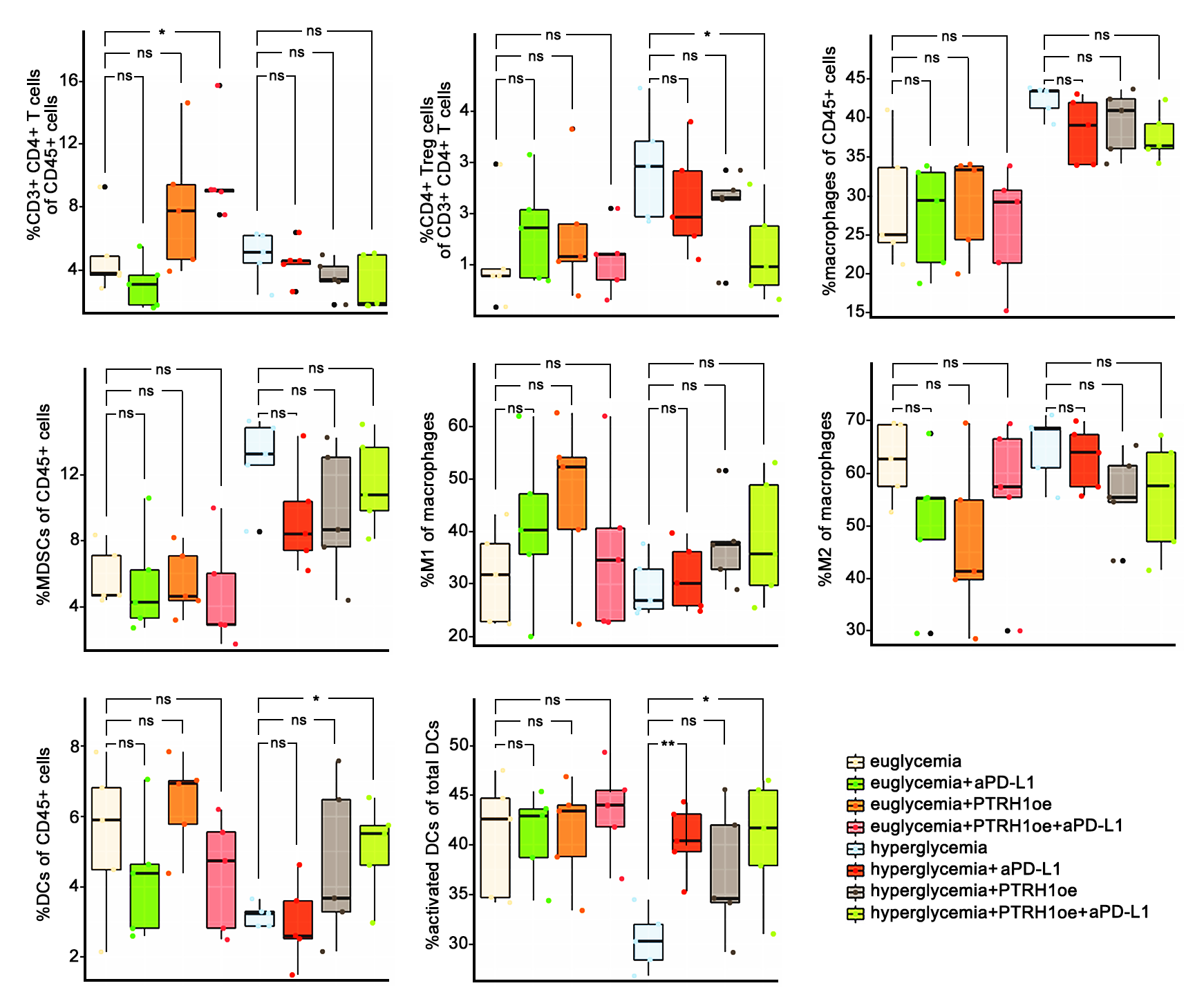

Supplement: Supplementary file 12 — Additional file 12: Figure S12 Flow cytometry analysis of the infiltration of immune effectors (CD4+ T cells, MDSCs, macrophages and DCs) in the orthotopic tumors in immune-competent C57BL/6 mice in different groups. [file 12967_2023_4302_MOESM12_ESM.tif]

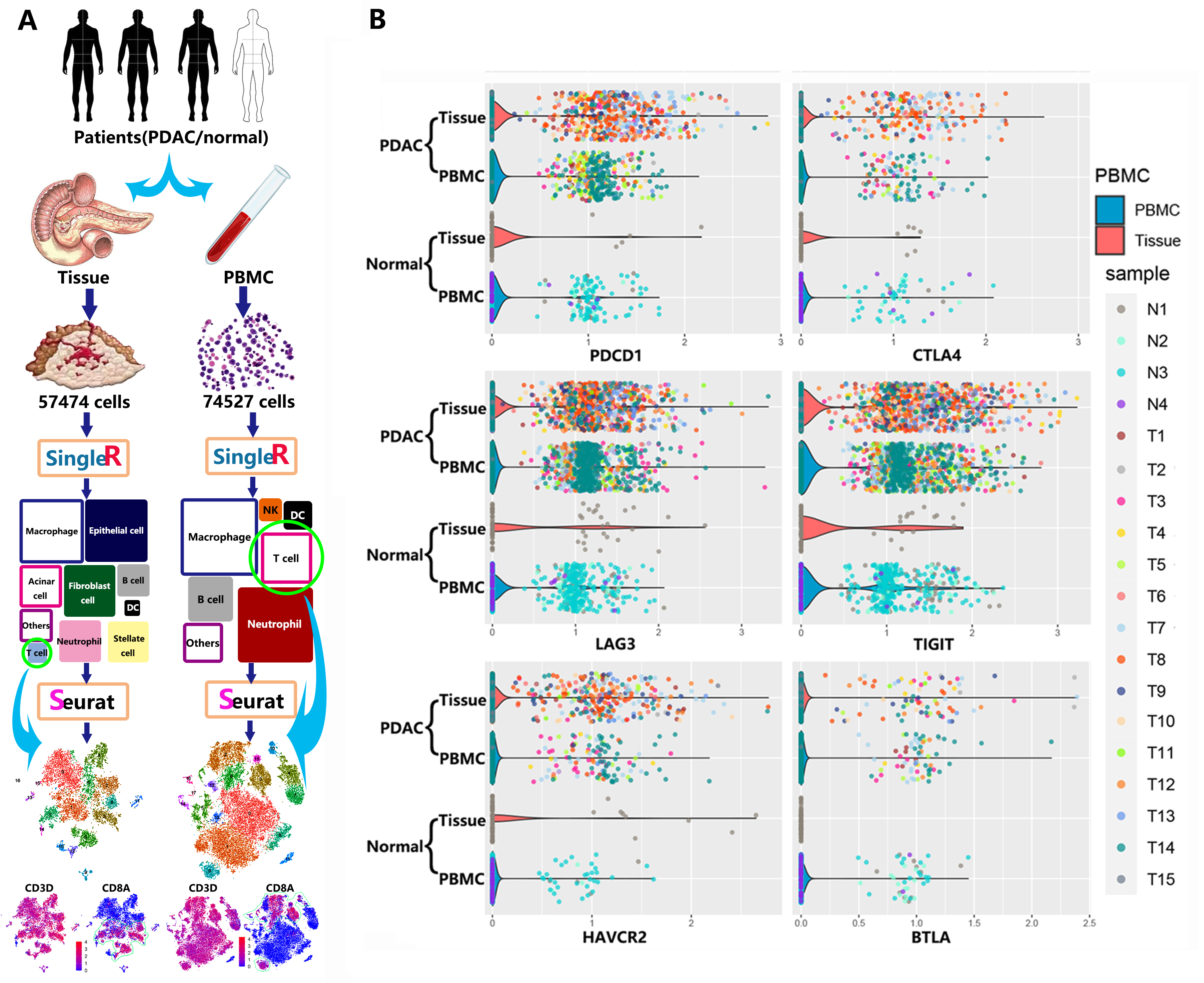

Supplement: Supplementary file 13 — Additional file 13: Figure S13 Immune checkpoints expression on CD8+ T cells in PDAC TME and peripheral blood. A Processing of identification of CD8+ T cells. B scatter plot depicting the differential level of several immune checkpoints on CD8+ T cells in PDAC TME and peripheral blood. [file 12967_2023_4302_MOESM13_ESM.tif]
